# Multidisciplinary Contributions and Research Trends in eHealth Scholarship (2000-2024): Bibliometric Analysis

**DOI:** 10.2196/60071

**Published:** 2025-06-16

**Authors:** Lana V Ivanitskaya, Dimitrios Zikos, Elina Erzikova

**Affiliations:** 1 Health Administration Division, School of Health Sciences The Herbert H and Grace A Dow College of Health Professions Central Michigan University Mount Pleasant, MI United States; 2 Department of Healthcare Management and Leadership Texas Tech University Health Sciences Center Lubbock, TX United States; 3 School of Communication, Journalism and Media College of the Arts and Media Central Michigan University Mount Pleasant, MI United States

**Keywords:** electronic health, eHealth, conceptual model, bibliometric analysis, VOSviewer, telemedicine, telehealth, virtual care, virtual health, virtual medicine, remote consultation, mobile health, mHealth, mobile application, app, application, smartphone, digital, digital health, digital technology, digital intervention, artificial intelligence, AI

## Abstract

**Background:**

Fueled by innovations in technology and health interventions to promote, restore, and maintain health and safeguard well-being, the field of eHealth has yielded significant scholarly output over the past 25 years.

**Objective:**

This study aims to offer a big picture of research developments and multidisciplinary contributions to eHealth that shaped this field up to 2024. To that end, we analyze evidence from 3 corpora: 10,022 OpenAlex documents with eHealth in the title, the 5000 most relevant eHealth articles according to the Web of Science (WoS) algorithm, and all available (n=1885) WoS eHealth reviews.

**Methods:**

Using VOSviewer, we built co-occurrence networks for WoS keywords and OpenAlex concepts. We examined clusters, categorized terminology, and added custom overlays about eHealth technologies, stakeholders, and objectives. A cocitation map of sources referenced in WoS reviews helped identify scientific fields supporting eHealth. After synthesizing eHealth terminology, we proceeded to build a conceptual model of eHealth scholarship grounded in bibliometric evidence.

**Results:**

Several research directions emerged from bibliometric networks: eHealth studies on self-management and interventions, especially in mental health; telemedicine, telehealth, and technology acceptance; privacy, security, and design concerns; health information consumers’ literacy; health promotion and prevention; mHealth and digital health; and HIV prevention. Conducted at the individual, health system, community, and society levels, eHealth studies focused on health and wellness across the human lifespan. Keywords such as *internet* (mean publication year 2017), *telemedicine* (2018), *telehealth* (2018), *mHealth* (2019), *mobile health* (2020), and *digital health* (2021) were strongly linked to literature indexed with *eHealth* (2019). Different types of eHealth apps were supported by research on infrastructures: networks, data exchange, computing technologies, information systems, and platforms. Researchers’ concerns for eHealth data security and privacy, including advanced access control and encryption methods, featured prominently in the maps, along with terminology related to health analytics. Review authors cited a wide range of medical sources and journals specific to eHealth technologies, as well as journals in psychology, psychiatry, public health, policy, education, health communication, and other fields. The *Journal of Medical Internet Research* stood out as the most cited source. The concept map showed a prominent role of political science and law, economics, nursing, business, and knowledge management. Our empirically derived conceptual model of eHealth scholarship incorporated commonly researched stakeholder groups, eHealth application types, supporting infrastructure, health analytics concepts, and outcomes.

**Conclusions:**

Drawing upon contributions from many disciplines, the field of eHealth has evolved from early studies of internet-enabled communications, telemedicine, and telehealth to research on mobile health and emerging digital health technologies serving diverse stakeholders. Digital health has become a popular alternative term to eHealth. We offered practical implications and recommendations on future research directions, as well as guidance on study design and publication.

## Introduction

### Background

The field of eHealth is about the use of digital technology in health care delivery, management, and education. In its definition, the World Health Organization (WHO) emphasizes the aspects of cost-effectiveness and secure use of information and communications technologies in support of health and health-related fields [1]. Expedited by the recent COVID-19 pandemic [2,3], multiple technologies that are broadly labeled as eHealth facilitate remote patient monitoring, improve access to medical services, and enhance efficiency in health care systems. For example, mobile health (mHealth) apps offer significant value to patients [4] by supporting data sharing with health care providers. This enables personalized care, promotes continuity of care, and enhances understanding of condition progression and treatment response during medical appointments. Artificial intelligence (AI), a recent advancement in eHealth, is poised to reshape medicine, improving the experiences of health care professionals and patients [5] through pattern recognition and generating insights that can improve diagnosis, treatment, and patient outcomes. These and other eHealth technologies enable patients to actively participate in their health care decisions and promote preventive care through personalized health information [6]. With the potential to streamline workflows and improve health care outcomes, eHealth leverages IT to transform access to and delivery of health care services [7].

This comprehensive bibliometric analysis examines the scholarly landscape of eHealth research over the past 25 years. We map research directions in this domain and the contributing scientific disciplines that have shaped the field. Bibliometric methods allow to quantitatively analyze published studies and their metadata to describe research output and to visualize intellectual structures and trends [8] in scientific domains of interest. The field of eHealth was the subject of bibliometric reviews; however, their scope was almost always limited to select technologies, regions, eHealth user experiences, or narrowly defined health and wellness goals.

Most bibliometric reviewers summarized literature subsets defined by eHealth user needs, such as promoting physical activity, healthy eating, and weight loss [9-12]; preventing substance use [13]; and providing e-mental health services during the COVID-19 pandemic [14]. In addition, bibliometric researchers reviewed digital technologies for health behavior change [15] and eHealth tools for anticoagulation management after cardiac valve replacement [16].

A distinct subset of bibliometric studies focused on eHealth and health informatics competencies [17], literacy [18], and information and communication technology use by individuals experiencing homelessness [19]. Region-specific bibliometric reviews demonstrated global interest in eHealth research: medical informatics and telemedicine in sub-Saharan Africa and BRICS (Brazil, Russia, India, China, South Africa) countries [20], eHealth research in Southeast Asia [21], and European funding of research on ambient assisted living [22].

Technology-centered bibliometric reviews assessed literature on technology adoption [23], telehealth [24], the internet of things [25], telemedicine in rural areas for cost-effective and sustainable health care [26], mHealth as a means of involving citizens and public agencies in cardiovascular disease prevention [12], and AI adoption by health care organizations [27].

Two broad-scope bibliometric reviews published in 2022 were dedicated to eHealth [28] and digital technologies [29]. The former study was limited to 2989 bibliometric records (2000-2021) that mentioned eHealth in titles; the latter included only 403 recent (2017-2021) publications. Other eHealth reviews published before 2022 did not include recent studies on evolving eHealth technologies, such as blockchain and AI [6,30].

### Objectives

To address gaps and limitations of past bibliometric reviews of eHealth, this study was designed with a broad chronological scope (2000-2024). It included recent publications from 2022 to 2024 from 2 different databases, a comparison of articles and reviews, and documents that mentioned eHealth not only in titles but also in abstracts or keywords. Our overarching aim was to examine a wide range of studies to provide a comprehensive overview of the eHealth research field, tracking its research directions, concept evolution, chronological developments, and multidisciplinary roots. A broad understanding of eHealth scholarship, as it developed over 25 years, is critical for informing policy makers, funders, researchers, educators, and practitioners about the dynamics of the field and thus supporting informed decision-making and advancing further research.

First, we endeavored to reveal research directions by analyzing the structure and contents of bibliometric networks. The network structures may help to identify distinct groupings of interrelated research topics. The network contents may provide clues about the stakeholder groups and their needs, which eHealth technologies were designed to support. We asked the following question: What research directions define the domain of eHealth? (research question [RQ1])

Second, we aimed to understand the chronological development of eHealth scholarship by examining publication trends across 2 document types: research articles and review articles. Their comparison might indicate areas that had accumulated a sufficient body of primary literature to warrant its synthesis. In addition, to gain insights into the maturation of research concepts and topics within the broader eHealth domain, we attempted to identify temporal lags between the emergence of active research areas and the publication of corresponding review articles summarizing those literatures. We posed the following question: How did eHealth scholarship—articles and reviews—develop over time? (RQ2)

Third, to offer a comprehensive account of eHealth as a multidisciplinary field, we attempted to document the disciplinary origins by mapping intellectual structures that have shaped the eHealth field. Our inquiry was guided by the following question: On what scientific fields does eHealth research build, as evidenced by cited sources and OpenAlex concepts that tag eHealth articles? (RQ3)

## Methods

### Ethical Considerations

This bibliometric study does not involve any human subject as the term is defined at CFR 46.102(e)(1). It uses bibliographic data about academic publications from publicly accessible databases, adhering to the principles of open science. Therefore, ethical concerns related to informed consent and privacy are not applicable and we did not seek an ethics review board assessment.

### Overview of Data Sources

We retrieved and screened 2 sets of Web of Science (WoS) records with eHealth or e-Health in titles, abstracts, or keywords: (1) 5000 most relevant articles, according to the WoS ranking algorithm; and (2) a nonoverlapping collection of 1885 WoS eHealth reviews written in English since 2000.

**Figure 1:**
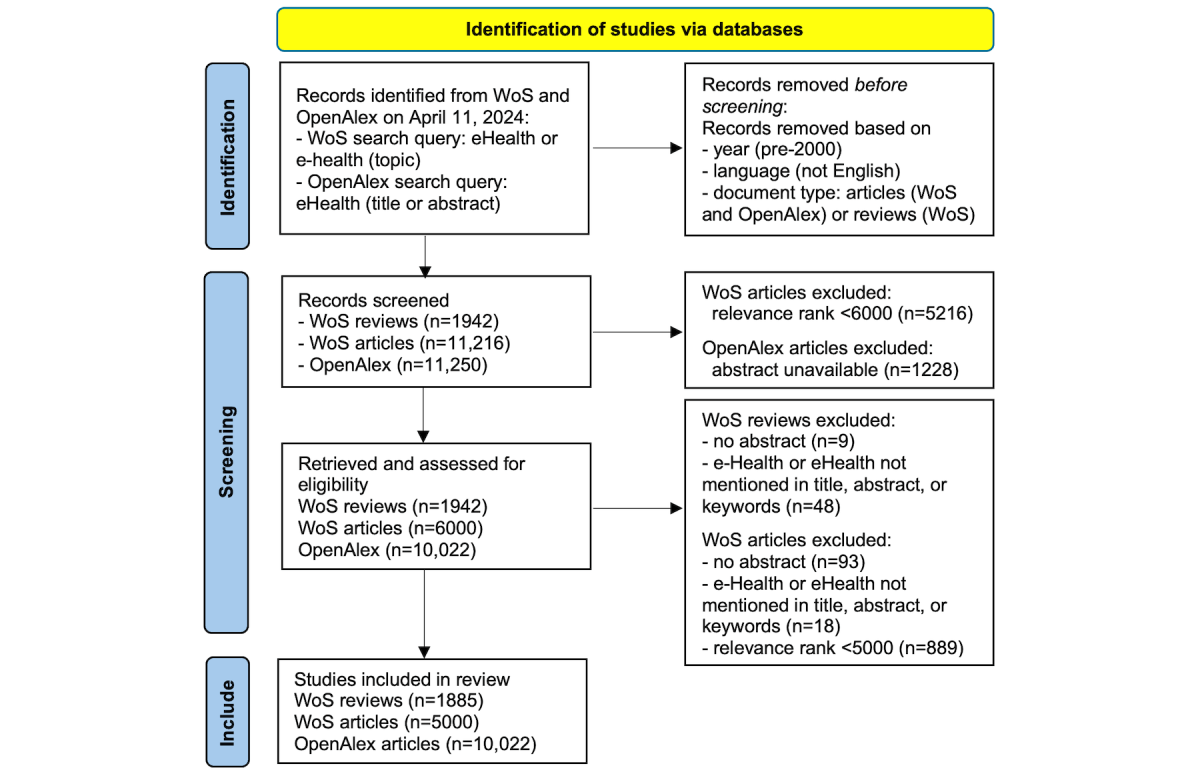
PRISMA diagram for eHealth publications included in this review. WoS: Web of Science.

Figure 1 shows the study identification process as a PRISMA (Preferred Reporting Items for Systematic Reviews and Meta-Analyses) diagram, including search queries performed in WoS and Open Alex databases; initial removal of records based on year, language, and document type; and subsequent record screening choices guided by RQs and database limitations.

The hyphenated search term, e-Health, produced both relevant and irrelevant records. Any word ending with *e* before the word *health* was counted as e-Health, prompting manual screening of WoS records. Not knowing exactly how many records would be screened out, we oversampled WoS articles. Only 5000 WoS articles with the highest relevance ranks were retained after screening. To extend our WoS findings, we also obtained 10,022 OpenAlex articles with eHealth in their titles or abstracts. OpenAlex search query was limited to eHealth to avoid potential issues with the hyphenated search term.

### Bibliometric Approach

We built and analyzed the following types of bibliometric networks or maps in VOSviewer [31] designed by researchers from the Center for Science and Technology Studies at Leiden University in the Netherlands: keyword co-occurrence networks for WoS datasets and a concept co-occurrence network for an OpenAlex dataset. The contents of networks were examined to identify eHealth research directions, conceptualized as eHealth technologies, stakeholders, and their needs. Keywords are controlled vocabulary used by the authors and WoS database managers to index studies. They differ from concepts that OpenAlex assigns to the majority (85%) of published works in its database. OpenAlex uses a hierarchical system of approximately 65,000 concepts, each linked to a Wikidata ID, to tag scientific publications with ≥1 concepts that are assigned based on the contents of the title, abstract, and the title of the host venue [32]. Concepts could add value above and beyond keywords because OpenAlex designers hierarchically organized concepts such as a family tree, starting with 19 major categories that branch out into discipline-relevant concepts [32].

Bibliometric networks consist of nodes, for example, keywords, concepts or cited journals, and the lines that link them [31]. The links represent relationships between nodes. For example, the more publications in our collection are indexed with the same keywords (or tagged with the same concepts), the stronger they would be linked and the closer they would be located to each other in our maps. Node size indicates the frequency of a specific keyword or concept—the number of documents that are indexed or tagged with it. Moreover, related nodes are grouped into clusters. Network overlays are information layers that highlight select keywords in red, such as those related to eHealth objectives, while the remaining keywords are shown in blue. Overlays help to contextualize the meaning of individual keywords through spatially close and linked nodes, which show keyword co-occurrences and interconnected research domains.

An abstract review was performed on multiple occasions to decipher ambiguous keywords or concepts or to identify examples of studies relevant to our main findings.

### Data Analyses

To answer RQ1, we analyzed network clusters—groups of co-occurring keywords or concepts that commonly reflect thematically distinct research directions [33,34]. A comparative analysis of 2 keyword co-occurrence networks was done. The network built for articles most relevant to eHealth, as determined by the WoS ranking algorithm, was compared to the network for all available eHealth reviews from WoS. Specifically, we compared clusters that imply research directions and node sizes, indicative of similarities and differences in research directions pursued by article authors versus review authors. In both maps, we assessed nodes with the strongest links to eHealth to reveal terminology at the heart of this research domain.

We used binary 0 and 1 coding to highlight keywords about groups involved with eHealth (who); health conditions, needs, or care settings addressed by eHealth interventions (what); and eHealth technologies or technology-related keywords (how). To estimate reliability, a second trained coder independently identified technology-related keywords from a list of 677 keywords selected for mapping, achieving a high level of agreement (κ=0.96; 95% CI 0.93-0.98; *P*<.001). Additional keyword coding was done as we developed a conceptual model of eHealth research. Binary codes were assigned to technology keywords based on their relevance to eHealth umbrella terminology or eHealth applications, objectives, infrastructure, data security and privacy, and health analytics. We added the aforementioned codes to a scores file in VOSviewer to display them as custom overlays to the keyword map for WoS articles.

RQ2 was answered by contrasting network overlays to draw conclusions about publication recency for articles and reviews. We also computed mean publication years for groups of keywords that characterized eHealth application types.

Evidence for RQ3 came from the scientific literature behind eHealth reviews, which were assessed using a cocitation map. In this network type, the nodes correspond to journals and other cited sources. The relationships between journals are defined based on the frequency with which they are jointly referenced or “cocited” within the bibliographies of eHealth literature reviews. The larger the node size, the more frequently the journal was cited by the authors of eHealth reviews. We chose reviews for this analysis because their bibliographies tended to be the most comprehensive and focused on well-researched eHealth aspects.

A concept co-occurrence network for OpenAlex eHealth studies was used to gather additional evidence about the multidisciplinary nature of eHealth to answer RQ3. In an OpenAlex concept map, many nodes are represented by discipline-relevant concepts nested within major categories, both of which are relevant to answering RQ3. Tagging the greatest number of eHealth studies, major categories would be the largest nodes in this map, whereas the smallest nodes would be specific topics relevant to our understanding of technologies researched by eHealth scholars. Similar to the network of WoS article keywords, we enhanced the OpenAlex concept network with custom overlays highlighting technologies, health topics (physical health, illness, wellness, and mental health), and other concept characteristics such as risk (eg, security) and money (eg, economics). Concept attributes were first coded using linguistic inquiry and word count (LIWC)–22, a computational linguistics program, and then converted to binary scores (code 0, or “not present,” was assigned to LIWC scores of 0, and code 1 was assigned to all other LIWC scores). The binary scores were manually verified and refined before being added as new overlay scores to the VOSviewer map file, in addition to mean publication year and normalized citations overlays.

To help readers follow our map interpretations, we used italics to indicate specific network nodes, whether they are WoS keywords, cited journals, or OpenAlex concepts. Unless noted otherwise, we consistently listed nodes based on the number of articles they represented, from high to low.

## Results

### eHealth Research Directions: Articles

**Figure 2:**
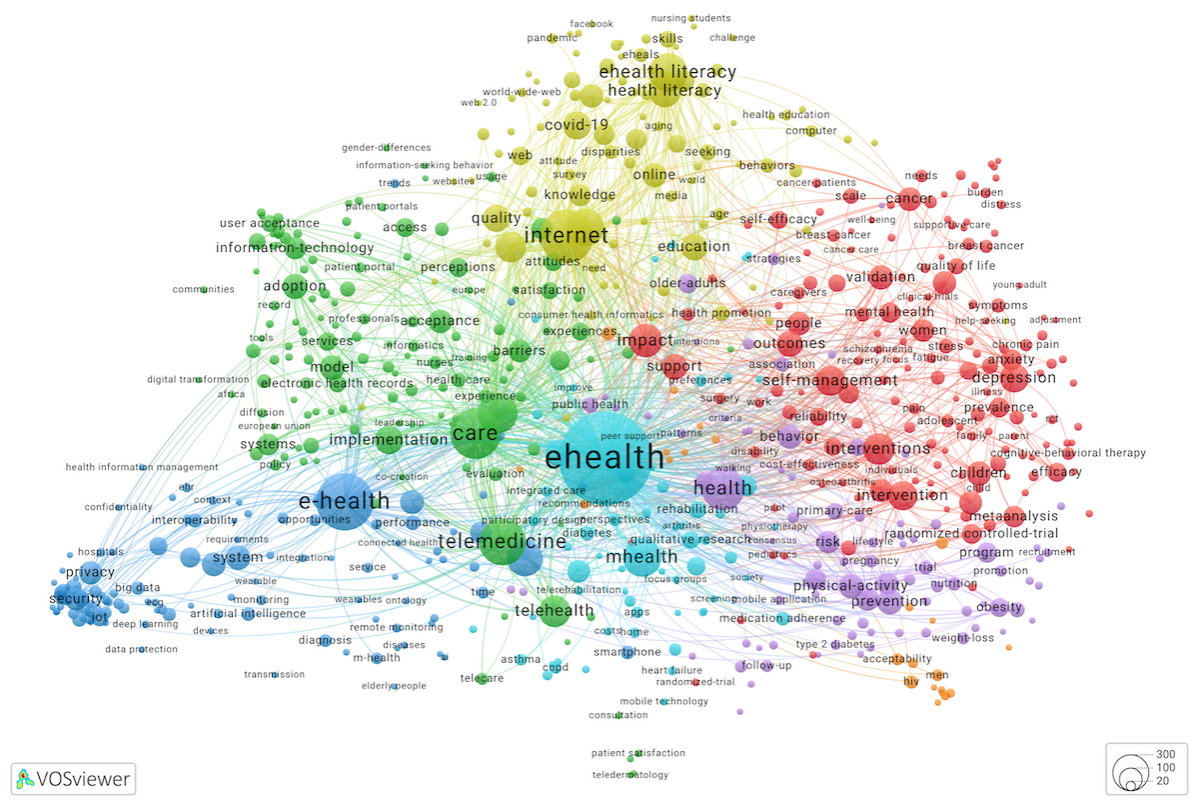
Keyword co-occurrence network (cluster map) for 5000 eHealth articles. Keywords that occur ≥10 times were mapped. An interactive map is available from Leiden University’s VOSviewer Online application.

In Figure 2 [35], we presented a keyword co-occurrence cluster map. Color-designated clusters are thematically linked groups of keywords derived from WoS articles. We provided a URL for an interactive map where the number of articles indexing each keyword can be explored, as well as keyword interconnections. The more frequently 2 keywords co-occur across multiple articles, the more likely they are to be located near each other, within the same cluster, and linked. The map shows 677 keywords and 1000 strongest links. Alternately spelled nodes *eHealth* and *e-Health* had the strongest co-occurrence link. In addition, the keyword *eHealth* was strongly linked to 9 other keywords: *telemedicine, mHealth, internet, digital health, self-management, mobile health, intervention, telehealth,* and *depression*.

Next, we summarized clusters by categorizing their most frequently occurring keywords in Multimedia Appendix 1 to identify stakeholders, care needs or settings, and eHealth technologies. Cluster 1 (shown in red in Figure 2) encompassed thematically diverse nodes related to eHealth with a centrally positioned *self-management* keyword indexing 217 articles, the third highest occurring keyword after *impact* and *interventions*. Abstracts that mentioned “self-management” suggested that the authors defined it as an oversight of one’s own health conditions, for example, to cope with a chronic disease by reducing anxiety, fatigue, or depression or to prevent negative health outcomes. Mental health (*depression, anxiety, psychological distress, schizophrenia,* and *cognitive-behavioral therapy*), cancer, and pain-related keywords were particularly prominent in this cluster. The cluster had keywords that described study populations: young people, survivors of cancer, and caregivers. Article authors reported eHealth intervention technologies—web based and mobile applications—used for assessment, reporting of symptoms and adverse events, cognitive interviewing, and supporting self-management goals, for example, by generating and communicating self-management actions. Together, the keywords *intervention* and *interventions* are indexed in 863 studies.

Keywords are listed in the order of their occurrence counts, from high to low, excluding the keywords used to index <10 articles. All overlays to the Figure 2 map are listed in Multimedia Appendix 1 and can be explored interactively.

Cluster 2 (green) keywords were dedicated to *telemedicine* and *telehealth* as well as health organizations’ electronic record systems (eg, *electronic health records*) used for storing information that is accessed, used, and documented during a telehealth session. This cluster’s keywords mentioned eHealth stakeholders who were patients, different health care professional groups, health leaders, and communities. Specifically, telemedicine was researched as a means of building community capacity and communities of practice. In rural communities, telemedicine connected remote populations to health care professionals, strengthening local health systems. Geographically dispersed health care professionals could improve their medical practice through telemedicine-enabled knowledge sharing within communities of practice. This cluster also prominently featured nodes related to the *acceptance* and *adoption* of eHealth technologies. Abstracts that mentioned *Technology Acceptance Model* (TAM)*,* or Unified Theory of Acceptance and Use of Technology (UTAUT), referred to stakeholder reactions to telehealth technologies and their impact on patient–health care professional relationship, with an emphasis on improved *access* to care and *patient empowerment*, *patient engagement*, and *patient participation*.

Keywords in cluster 3 (dark blue) were especially focused on 3 technological aspects of eHealth, namely, eHealth technology infrastructure, data security and privacy, and health analytics. Keywords relevant to the eHealth technology infrastructure were, for example, *internet, online, internet of things, cloud computing, blockchain, information-systems, interoperability, smartphone, cloud,* etc. The second group included *security, blockchain, authentication, encryption, access control, cryptography, privacy protection, access-control*, and other data security and privacy considerations. The third group was about health analytics: *artificial intelligence, machine learning, big data, algorithm, algorithms, deep learning, data mining,* etc. The most frequent stakeholder keywords in cluster 3 were *management* and *hospitals*, in contrast to keywords related to patients in cluster 1 and health care professionals in cluster 2.

Cluster 4 (yellow) was about eHealth literacy, health information seeking, and concerns about misinformation and decision-making during the COVID-19 pandemic. This subset of studies focused on younger and older age groups, with a strong focus on students. With the help of eHealth tools and skill assessments for health education, researchers studied demographic and behavioral aspects of health information seekers who engage with web-based health information. They described their research using keywords such as *internet, eHealth literacy, social media, computer, digital health literacy, consumer health informatics, world-wide-web,* and *website*. This cluster also included the keywords *disparities* and *digital divide*.

The remaining 3 clusters contained the smallest number of keywords. Nodes in cluster 5 (purple) reflected the needs of adults and older adults related to physical activity and lifestyle changes aimed at preventing obesity, hypertension, cardiovascular disease, and diabetes. Researchers studied how these needs were addressed through eHealth interventions and mobile apps. Cluster 6 (light blue) keywords suggested a focus on eHealth, mHealth, and digital health applications, as well as telemonitoring, telerehabilitation, and communication technologies, for managing chronic diseases and medication adherence in older adults. The care types spanned primary care, rehabilitation care, home care, and integrated care. Finally, keywords in cluster 7 (orange) indexed research on eHealth interventions for HIV prevention among men who have sex with men.

While analyzing clusters, we found keywords that could be described as general or umbrella terms (*ehealth, e-health, technology, digital health, internet use*, etc) and more specific eHealth applications (*telemedicine, mhealth, telehealth, mobile health, electronic health record, telecare*, etc). In addition, we encountered many instances of keywords that shed light on eHealth objectives (*ehealth literacy, health literacy, communication, education, prevention, quality-of-life*, etc). Scattered across all clusters, eHealth objectives pertained to stakeholders’ health conditions, needs, or care settings. We coded these keyword groups, as well as subgroups about technology infrastructures, data security and privacy, and health analytics, to make them available as overlays (Multimedia Appendix 1).

### eHealth Research Directions: Reviews Compared to Articles

**Figure 3:**
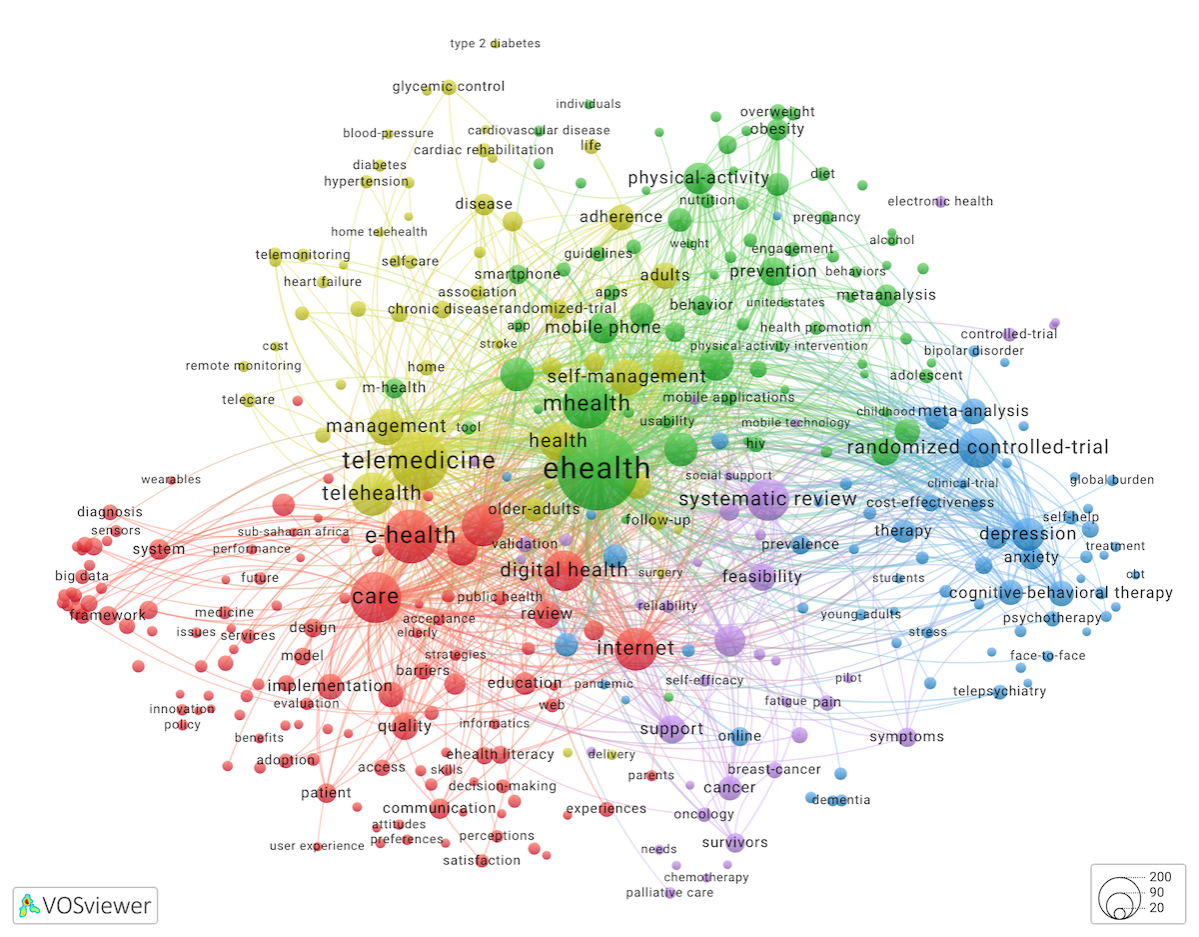
Keyword co-occurrence network (cluster map) for 1885 eHealth reviews. Keywords that occur ≥10 times were mapped. An interactive map is available from Leiden University’s VOSviewer application.

Figure 3 [36] shows a cluster map for reviews obtained from the WoS database. Cluster colors in Figures 2 and 3 were set automatically by VOSviewer based on the number of nodes in a cluster. Despite differences in cluster colors, many keywords, for instance, those related to mental health or obesity prevention, were grouped in similar ways in both maps. Out of 358 keywords that appeared in Figure 3, 318 (88.9%) were present in Figure 2. Similar to Figure 2, the node *eHealth* in Figure 3 was strongly linked to nodes *mHealth, telemedicine, digital health, telehealth,* and *internet*.

A close examination of a subset of 318 keywords that appeared in Figures 2 and 3 revealed differences in eHealth topics covered by articles versus reviews (Table 1). A delta of z-scored keyword occurrence counts for keywords used to index reviews versus articles was used as an indicator of research focus for the 2 document types. We asked which eHealth topics were more or less likely to be covered by eHealth reviews as compared to eHealth articles? Several patterns emerged when we analyzed differences (Δ>0.5 SD) [37].

Similar to reviews conducted in other health disciplines [38], eHealth review authors attempted to summarize experimental research. A *randomized controlled trial* keyword indexed a disproportionately greater share of reviews than articles. Second, review authors favored studies on telemedicine, telehealth, digital health, and mHealth. Feasibility studies were also a likely subject of literature reviews.

**Table 1:** Top keywords indexing eHealth articles, by cluster, compared to keywords indexing eHealth reviews.

eHealth articles (Figure 2)		eHealth reviews (Figure 3)
Cluster number (color^a^) and name	10 most frequent keywords^b^	Keywords^b^ more (+) and less (–) likely used to index reviews, as compared to articles, in SD units
1 (Red): self-management and interventions	*impact, interventions, self-management, intervention, depression, outcomes, support, children, validation,* and *cancer*	More likely: randomized controlled-trial (+1.9) and feasibility (+0.7) Less likely: impact (–1.0), cognitive-behavioral therapy (–0.7), social support (–0.7), and self-efficacy (–0.6)
2 (Green): telemedicine, telehealth, telecare, and technology acceptance	*care, telemedicine, technology, telehealth, implementation, adoption, model, acceptance, barriers,* and *information-technology*	More likely: telemedicine (+1.2) and telehealth (+1.1) Less likely: adoption (–1.2), acceptance (–1.2), barriers (–0.8), implementation (–0.7), trust (–0.7), usability (–0.7), and user acceptance (–0.7)
3 (Dark blue): eHealth technology, including privacy, security, and design	*e-health, management, system, health care, framework, privacy, security, design, challenges,* and *healthcare*	Less likely: e-Health (–2.2), privacy (–0.9), security (–0.8), design (–0.6), internet of things (–0.5), and cloud computing (–0.5)
4 (Yellow): eHealth literacy	*internet, ehealth literacy, information, health literacy, communication, covid-19, quality, education, online,* and *health information*	Less likely: internet (–7.5), ehealth literacy (–3.0), information (–2.2), health literacy (–1.9), communication (–1.5), health information (–1.2), literacy (–1.0), covid-19 (–0.9), education (–0.8), internet use (–0.8), older adults (–0.6), quality (–0.5), and skills (–0.6)
5 (Purple): health promotion and disease prevention	*health, prevention, physical-activity, behavior, adults, risk, physical activity, exercise, program,* and *older-adults*	No differences greater than +0.5 or −0.5 SD were observed
6 (Light blue): mHealth^c^ and digital health	*ehealth, mhealth, digital health, mobile health, mobile phone, primary care, chronic disease, qualitative research, rehabilitation,* and *diabetes*	More likely: digital health (+1.4), mhealth (+1.0), mobile health (+0.8), and mobile phone (+0.7) Less likely: ehealth (–7.5) and primary care (–0.8)
7 (Orange): HIV prevention	*decision-making, hiv, united-states, acceptability, men, implementation science, recommendations, gay, hiv prevention,* and *intervention development*	No differences greater than +0.5 or −0.5 SD were observed

^a^Cluster colors refer to Figure 2, a keyword co-occurrence network for 5000 eHealth articles, and an interactive map available on the VOSviewer website.

^b^Keywords from Figure 2 are italicized.

^c^mHealth: mobile health.

Other keywords salient in the eHealth article map did not receive much attention from review authors. Two findings that stood out the most were (1) few reviews of *eHealth* (or *e-Health*) literature, a research domain this study was designed to address; and (2) a disproportionately small number of reviews on eHealth literacy relative to the number of articles in this area. In addition, reviews somewhat underrepresented studies on eHealth technologies indexed with keywords *privacy* or *security* and issues of eHealth technology adoption, such as *barriers*, *usability*, and *user acceptance*. Some mental health keywords, for instance, eHealth applications of *cognitive-behavioral therapy* or those related to *social support* and *self-efficacy,* were more frequently used to index articles than reviews. These underreviewed topical areas may be considered by systematic review authors interested in eHealth.

### Publication Recency

**Figure 4:**
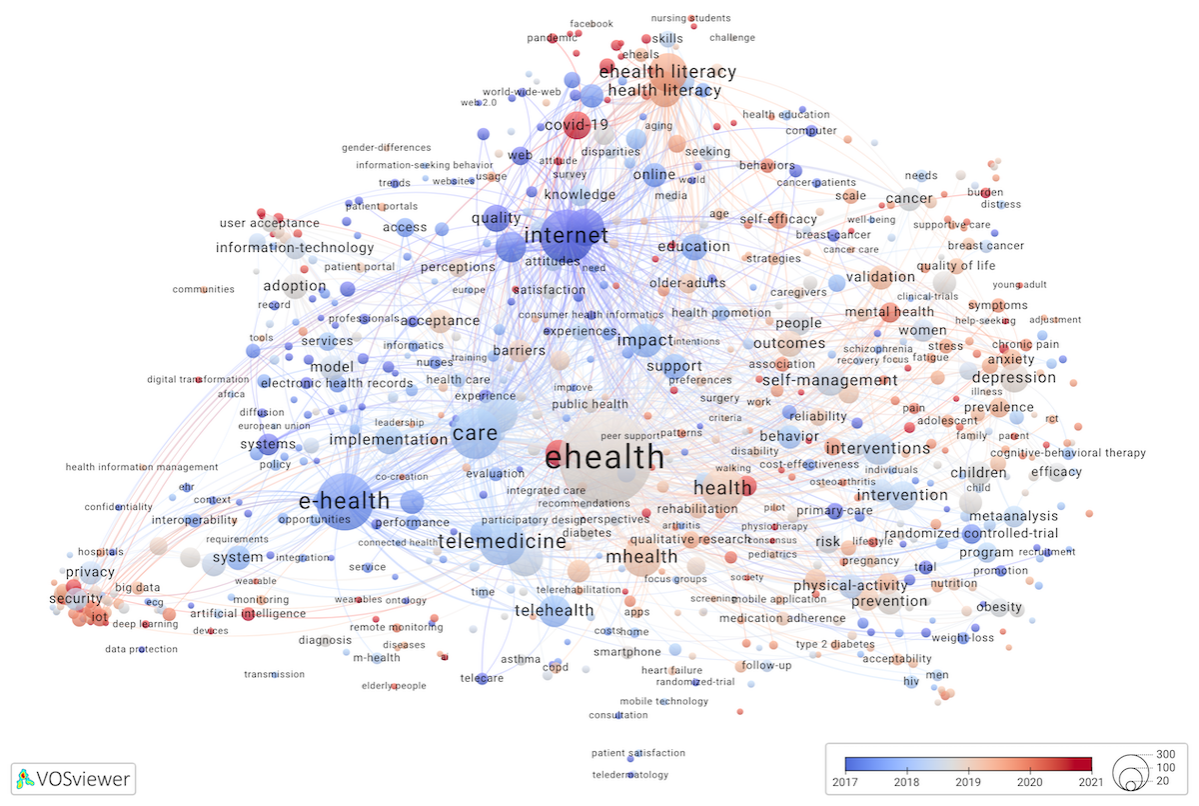
Publication recency overlays to maps in Figures 1 and 2: keywords indexing articles (top) and reviews (bottom). Interactive overlays (articles and reviews).

To answer RQ2, a comparison of mean publication years overlays for eHealth articles and reviews was used to identify trends in empirical research production and its subsequent synthesis. Figure 4 [39,40] shows publication recency overlays for eHealth articles and reviews.

Figure 4. Publication recency overlays to maps in Figures 1 and 2: keywords indexing articles (top) and reviews (bottom). Interactive overlays [39] (articles) and [40] (reviews).

Both map legends range from 2017 (blue) to 2021 (red) and are centered around 2019 (gray color). In the top overlay of Figure 4 with keywords for articles, *eHealth* was most strongly linked to *telemedicine* (mean publication year for all articles indexed with the keyword=2018) and *mHealth* (2019), followed by *internet* (2017), *telehealth* (2018), *mobile health* (2020), and *digital health* (2021). Mean publication years were most recent (2021-2022) for eHealth articles indexed with *Covid-19* or *pandemic, mindfulness, wearables, digital health, deep learning* and *blockchain, burden*, and *artificial intelligence*. Some of the same keywords (*deep learning, Covid-19*, and *artificial intelligence*) also represented the most recent (2021-2022) collections of reviews, in addition to the following keywords: *men, sedentary behavior, internet of things (iot), fatigue,* and *patient-reported outcomes*.

Excluding methods-related keywords, keywords with the oldest mean publication years (2012-2016) represented eHealth articles on *telepsychiatry, computer, web, information technology, ethics, weight loss, medical informatics, breast-cancer,* and *primary-care*. In addition to *health information technology and medical informatics*, the oldest reviews (2016-2017) were indexed with keywords *computer, health communication,* *smoking-cessation, telecare, records, user acceptance, electronic medical records,* and *internet use*. Importantly, e-Health consistently indexed older publications in both maps, as compared to eHealth, which is a welcome terminology standardization trend given the difficulties we encountered while retrieving e-Health publications.

Calculated across 318 keywords that appeared in Figures 2 and 3, the mean publication year was 2018.77 for *eHealth* articles and 2019.80 for *eHealth* reviews, a difference of about 12 months. The time gap between the mean publication date for all articles and all reviews indexed with *mHealth* was 8 months, M_356_=2019.47 for articles and M_303_=2020.10 for reviews. The time gaps were 11 months for studies indexed with *eHealth*, M_2089_=2019.08 and M_837_=2019.96, for articles and reviews, respectively; 15 months for *telemedicine*, M_522_=2018.32 and M_422_=2019.62; and 16 months for *telehealth*, M_242_=2018.07 and M_236_=2019.93.

### Multidisciplinary Contributions to eHealth Scholarship: Journal Names in Reference Sections of eHealth Reviews

**Figure 5:**
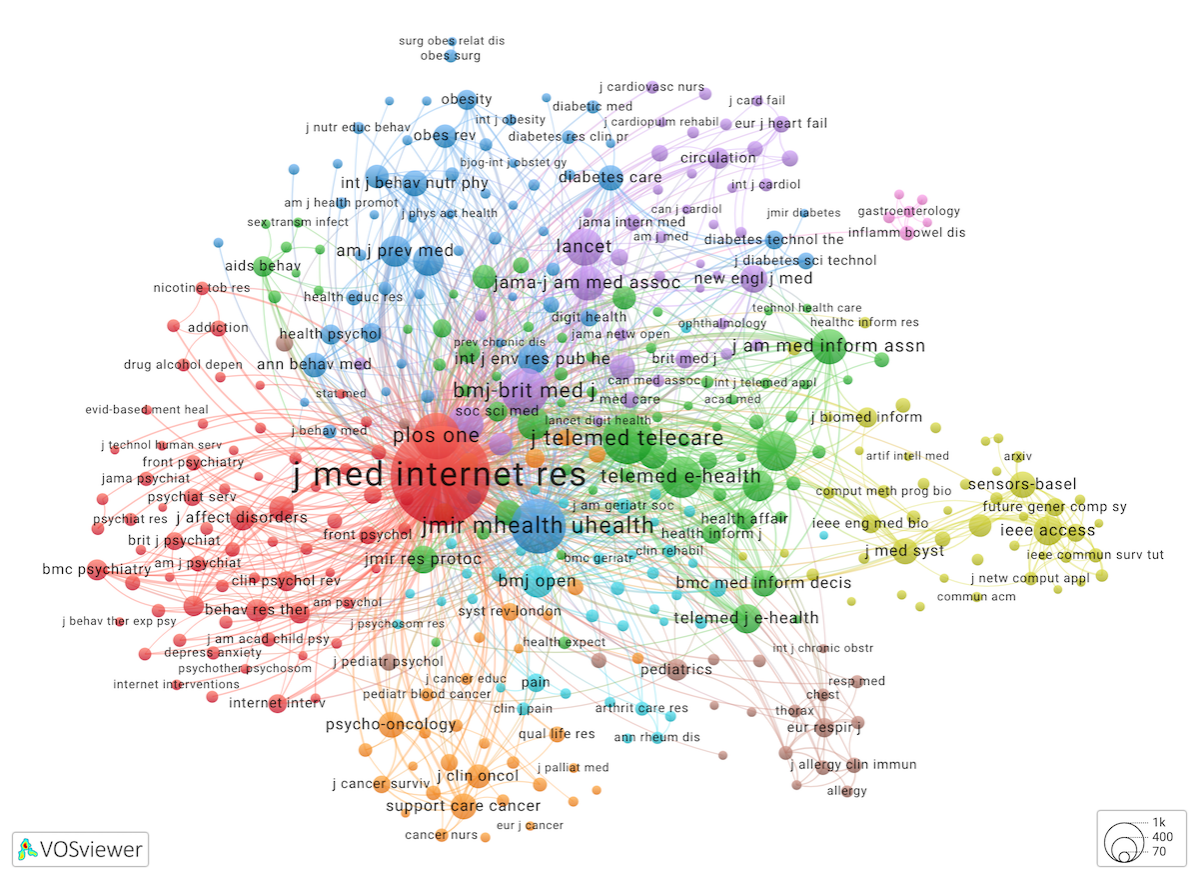
Cocitation network (cluster map) of sources for 1885 eHealth reviews. Sources that occurred ≥50 times in eHealth reviews’ reference lists were mapped. Link to an interactive map.

To answer RQ3, we analyzed multidisciplinary contributions using journal names that appear in reference sections of eHealth reviews (Figure 5) [41].

Figure 5. Cocitation network (cluster map) of sources for 1885 eHealth reviews. Sources that occurred ≥50 times in eHealth reviews’ reference lists were mapped. Link to an interactive map [41].

A 9-cluster model of journals contributing to eHealth reviews highlighted the leading role of the *Journal of Medical Internet Research*. It was cited the most, specifically 6329 times in 1884 reviews for which citation lists were available. It belonged to the largest cluster (cluster 1, red), with a large group of journals mostly dedicated to psychology and psychiatry.

In cluster 2 (green), the largest nodes were telemedicine, eHealth, and telecare journals, followed by journals in other disciplines—health informatics, public health, health services, medical education and health communication, clinical practice, HIV and AIDS research, and health care policy. Interestingly, we did not observe journals specializing in social media in this or any other cluster, given social media keywords observed in Figures 2 and 3.

Cluster 3 (dark blue) encompassed mHealth and ubiquitous health content (*JMIR mHealth and uHealth*), followed by cited sources in the fields of preventive medicine and public health; nutrition, obesity, and exercise; behavioral medicine and health psychology; and diabetes and endocrinology, among other disciplines.

Cluster 4 (yellow) was unique in that its sources were less likely to be cocited with sources from other clusters. Journals in cluster 4, related to sensors, AI, and health informatics, focused on IT, computing, health care, and biomedical topics. An interdisciplinary journal, *Nature*, was also in this cluster, a distant node with stronger cocitation ties to medical sources than most computing journals in this cluster.

Cluster 5 (purple) included journals in general and internal medicine, cardiology and cardiovascular medicine, epidemiology, and other specialized medical fields. Several leading medical journals (*The* *BMJ, The Lancet, JAMA: Journal of the American Medical Association*, and *The New England Journal of Medicine*) were among the largest nodes in this cluster.

In addition to general medical research sources, cluster 6 (light blue) had journals on pain, digital medicine, geriatrics and aging, rehabilitation and disability, rheumatology, and neurology. Cluster 7 (orange) was dedicated to cancer and oncology journals and journals in related health care fields, including psycho-oncology, palliative care and symptom management, nursing in oncology, quality of life and patient outcomes, cancer education, nursing, and palliative care.

Most journals in cluster 8 (brown) belonged to either respiratory medicine and allergology or pediatrics and adolescent medicine, confirming our earlier findings about eHealth interventions for this age group. Finally, cluster 9 (pink) consisted of gastroenterology journals, particularly those focusing on inflammatory bowel diseases and related conditions. It is important to note that some fields, such as nursing, were represented by journals in many clusters.

### Multidisciplinary Contributions: A Concept Map of eHealth Studies From OpenAlex

**Figure 6:**
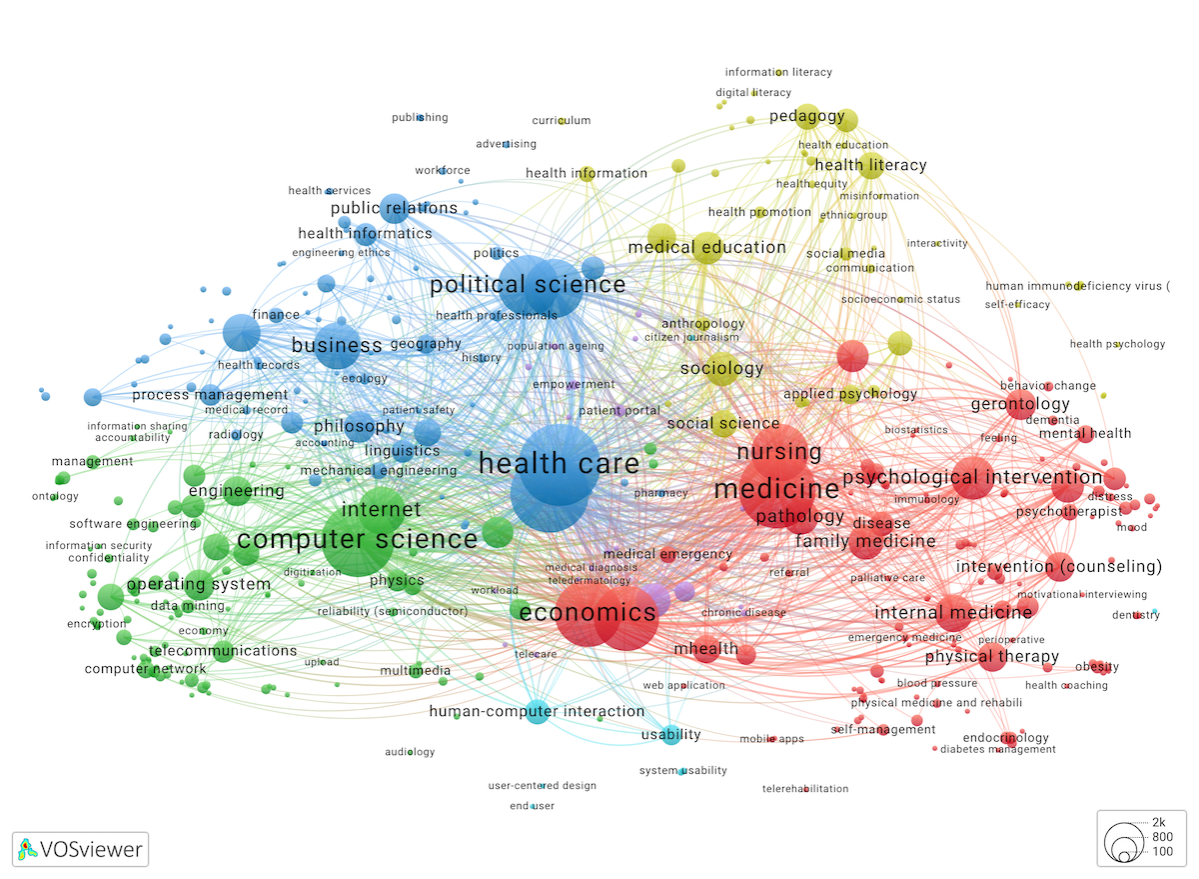
Concept co-occurrence network (cluster map) for 10,022 eHealth articles from OpenAlex. Concepts that occur ≥20 times were mapped. An interactive map is available from Leiden University’s VOSviewer application.

In addition to a cited journals analysis, we gathered evidence of multidisciplinary contributions directly from a large corpus of eHealth articles in OpenAlex, which were tagged with ≥1 concepts. Concepts reflect disciplines, theories, methods, and other abstract ideas. We developed a custom thesaurus to select discipline-revealing concepts for the map in Figure 6 [42]. Specifically, after removing most methods and statistics-related concepts (eg, sample or odds ratio), geography, and general ideas (eg, work) and merging synonymous concepts, we mapped the remaining 392 concepts representing disciplines and ideas relevant to eHealth. Each mapped concept occurred at least 20 times.

Figure 6. Concept co-occurrence network (cluster map) for 10,022 eHealth articles from OpenAlex. Concepts that occur ≥20 times were mapped. An interactive map is available from Leiden University’s VOSviewer application [42].

Figure 6 validated our earlier journal-level findings from Figure 5, confirming eHealth research connections to health care services, medicine, psychology, public health, education, and computer science. It also added to our understanding of the multidisciplinary nature of eHealth research by highlighting the prominent role, according to node size, of political science and law, economics, business, and knowledge management. The strongest connections with eHealth were observed for *medicine* and *nursing* and *computer science*, followed by *economics* and *economic growth, political science*, and *law*. While these concepts were most central to eHealth, numerous other fields, ranging from human-computer interaction and engineering to philosophy and linguistics, contributed to eHealth scholarship. In Figure 6 network, eHealth had the strongest links to *internet* and *intervention*, followed by the 4 concepts strongly connected to eHealth in Figures 2 and 3: *telemedicine, mHealth, digital health,* and *telehealth*.

In Table 2 [43-51], we contextualized our cluster-based findings with map overlays, examining how study attributes and concept characteristics were distributed across the map depicted in Figure 6. Links to an interactive map with overlays appear in Multimedia Appendix 1.

**Table 2:** Overlays to Figure 6 map: overlay name, interactive map link, definition, and summary.

Overlay name, interactive map link, and definition	Summary of findings
Publication year [43]: mean year for all articles represented by a concept	Overall, older publications appear on the left side of the map (business, engineering, law, political science, and many computer science concepts), while more recent publications (medical and health disciplines and human-computer interaction) are on the right. Notable exceptions in cluster 2 are as follows: *edge computing, deep learning, enhanced data rates for GSM evolution, audiology*, and *blockchain* with 2020 to 2021 mean publication years.
eHealth technology or related concept [44]	Most technology concepts fell under computer science and engineering. Health literacy ideas (eg, *digital divide*) and *social media* tended not to co-occur with other technology concepts.
eHealth objective: a concept related to desired outcomes or goals [45]	Objectives were widely spread across clusters. They fell into the categories of health care services (access, safety, and quality); supporting health care professionals; fostering sustainable and efficient health systems; encouraging collaboration and communication; promoting public health; enhancing user experience, empowerment, and engagement; and safeguarding data and information security.
Health issues or field [46]: broadly defined concepts related to health and health disciplines, including illness, wellness, and mental health	A plethora of disciplines are concerned with disease, health, and wellness (from high to low node size): *medicine, psychology, internal medicine, pathology, family medicine, psychiatry, environmental health, gerontology, physical therapy, public health, clinical psychology, surgery, alternative medicine,* etc.
Illness (a concept specific to diseases and health conditions): [47]	Pathology, surgery, infectious diseases, and cancer concepts had the highest number of studies.
Wellness (a concept specific to health promotion and maintenance): [48]	Health literacy, alternative medicine, quality of life, self-management, and physical activity interventions had the highest count of studies.
Mental health (a concept related to cognitive, behavioral, and emotional well-being) [49]	Psychology and psychiatry concepts, especially those related to interventions, had the highest count of studies. Conditions included anxiety, dementia, distress, suicide ideation, insomnia, depression, and addiction.
Risk (a concept related to risk in technology or health domains) [50]	Risk reduction concepts were related to computer security (access control, information security, and cloud computing security) and engineering risk analysis. Other risk concepts included pandemic risks, poison control, patient safety, suicide prevention, adverse effects, vaccination, and injury prevention.
Economics and business (economics and business-related concepts or fields) [51]	Economics, economic development, business, and marketing concepts had the highest count of studies.

According to the mean publication year overlay, the nodes with the most recent mean publication year were misinformation (2022) and new technologies such as edge computing (2021), deep learning (2022), pandemic concepts (2021), and concepts about mental health and psychological well-being, including depressive symptoms, mental health literacy, insomnia, and loneliness, all of which had a mean publication year of 2021. Older eHealth articles were represented by studies classified by OpenAlex as computer science, engineering, business, health informatics, and public relations. Setting aside concepts not specific to eHealth technologies, telematics (2008), semantic web (2013), web service (2013), IT (2013), ubiquitous computing (2013), health IT (2014), information sharing (2014), cross-domain interoperability (2014), and informatics (2014) were the oldest technology-related concepts with the mean publication year prior to 2015. These findings assist in answering RQ2 about eHealth research development over time. Other overlays explained in Table 2 addressed concepts specific to eHealth technologies and objectives, as well as different aspects of health. In the subsequent sections, we have highlighted several points that are most pertinent to multidisciplinary contributions.

The technology overlay demonstrated that 34.9% (137/392) of OpenAlex concepts mapping eHealth were directly linked to technology, a clear indication that eHealth multidisciplinarity is only partially grounded in data sciences, engineering, and computer sciences. Interestingly, the *social media* concept (also represented as a keyword in Figures 1 and 2) stood out as an eHealth literacy technology with relatively weak co-occurrence relationships with most other eHealth technologies. We found some evidence of research on social media information campaigns and pandemic interventions, for example, a moderated Facebook group that brought together 200 health care professionals and >58,000 laypeople from Denmark to support an informed approach to following pandemic guidelines [38]. Nevertheless, our maps consistently depicted social media as a small domain, suggesting this research played a modest role in the eHealth literature we examined.

When OpenAlex map and WoS maps were created, VOSviewer calculated node scores indicative of mean citations and normalized citations. These analyses were outside of the study scope; therefore, we have included them in Multimedia Appendix 1.

## Discussion

### Principal Findings

In the sections that follow, we synthesize eHealth research directions by creating typologies of eHealth applications, other technologies, and their objectives. Furthermore, we explore groups of stakeholders involved in eHealth and systematize levels of research. Our synthesis lays the groundwork for an evidence-based conceptual model of eHealth, grounded in a continuous 25-year research effort.

To illustrate the provided typologies with examples, we attempted to give priority to publications labeled by WoS as “highly cited papers” because their selection procedure controls for the field of study and the distribution of citations over time [52]. When no highly cited WoS papers were available, we manually picked pertinent articles and reviews using topic relevance, citation counts, and recency as our selection criteria.

In this section, we also discuss our findings related to publication recency and multidisciplinarity. We conclude by stating limitations and implications for research and practice.

### Objectives of eHealth

Keywords and concepts from our maps offered insights into the intended uses for eHealth technologies. Research into clinical applications of eHealth technology was in support of patient monitoring, diagnosis, treatment, and rehabilitation, as well as patient– health care professional communication. Public health objectives were directed at improving health literacy, education, prevention, quality of life, and well-being. There were also organizational and societal objectives, which we discuss in the eHealth Stakeholders and Research Analysis Levels section that introduces eHealth research levels.

Consistent with prior research that emphasized consumer-oriented eHealth solutions, patient-centeredness, and ownership of one’s health as defining features of eHealth [7], we found strong evidence of eHealth research on fostering engagement, self-care, participation, and person-centeredness. The following keywords from Figure 2 indexed the highest number of studies: *self-management, self-efficacy, social support, engagement, satisfaction, participation, self-care, empowerment, motivation, patient empowerment,* and *involvement*. Self-management apps, for instance, were successfully used to facilitate medication adherence and adherence to treatment of noncommunicable diseases: cancer, respiratory diseases, cardiovascular diseases, and diabetes. In 1 study, an electronic health record–linked web-based system for reporting cancer symptoms dispensed automated advice for either self-management or medical attention [53]. Improvements in physical and psychological well-being were reported for patients with cancer who used the system. A 2022 systematic review of factors influencing mHealth app adherence, defined as following the intended program as designed, found 17 studies of mHealth apps for self-management of noncommunicable diseases with >15,000 participants [54]. The review showed 53.4% overall app adherence and 89.5% medication app adherence.

This study highlighted eHealth objectives from past studies as an overlay to the concept map to guide objective setting by future eHealth designers. Our objectives overlay and the keyword map can be used for brainstorming possible measures to set intervention user prerequisites (eg, health literacy, self-efficacy, and motivation), outcomes, and manipulation checks to evaluate internal validity. The concepts and keywords in these maps reflect established terminology, facilitating literature searches for measurement instruments and the replication of past studies.

### Technologies Supporting eHealth

#### The Keyword

*eHealth* served as a catch-all term, labeling research on a variety of technologies. Using information from our maps, we systematized terminology relevant to eHealth applications and their supporting technological infrastructures. Infrastructures of interest to eHealth researchers included networks, data exchange, computing technologies, information systems, and platforms.

The highest number of studies was observed for the internet, followed by the internet of things, cloud computing, blockchain technology for secure data exchange, smartphones, sensors, fog computing, and 5G technology. Other well-studied eHealth infrastructures were wireless sensor networks and body area networks that expanded the reach of eHealth applications, facilitating remote monitoring and health interventions over vast geographic areas. Hardware, from smartphones to specialized sensors in wearables, enabled data collection and health monitoring. In addition, eHealth scholars explored platforms such as websites, social media, and mobile apps relevant to eHealth.

Researchers’ concern for eHealth data security and privacy, for example, through advanced access control mechanisms and encryption techniques, was a prominent feature in our maps, as well as terminology related to health analytics and AI that supports it.

Applications of eHealth can be categorized as education and informatics, telehealth, mHealth, health record management, and wearables. Table 3 [54-73] summarizes eHealth application types with references to well-cited papers that illustrate relevant research. Calculated for studies indexed with different keyword groups, mean publication years approximate an eHealth application development timeline, from education and informatics (2016) to wearables (2021).

**Table 3:** Characteristics of eHealth Web of Science articles by eHealth application type.

Application type	Keywords from Figure 2	Publication year^a^, mean	Research examples: relevant articles and reviews listed in chronological order
Education and informatics	*e-learning* and *consumer health informatics*	2016.24	A framework for analyzing health-related webpages [55] Consumer health informatics review [56]
Telehealth	*telemedicine, telehealth, telecare, telemonitoring, telerehabilitation,* and *teledermatology*	2018.02	A systematic review of telemedicine reviews calling for controlled interventions with emphasis on the patient perspectives, economic analyses, and evidence of effectiveness [57] Telehealth and telemedicine during COVID-19 [58] Applications of telemedicine [59]
Health record management	*electronic health record, ehr, patient portal(s),* and *personal health record(s)*	2018.45	Clinical predictions using big datasets [60] A model for distributed personal health records [61] Use of blockchain to improve electronic health record sharing [62] Health equity in patient portal research [63] Outcomes of patient portals and personal health records [64]
mHealth^b^	*mhealth, mobile health, m-health, app(s), mobile application(s) mobile app(s),* and *health apps*	2019.74	Theory and policy implications of “average” mHealth app users—young, highly educated, and eHealth literate [65] mHealth application selection based on user satisfaction, functionality, usability, and information quality [66] Adoption of mHealth services in Bangladesh [67] mHealth apps for prevention or management of noncommunicable diseases [54]
Wearables	*Wearable(s)* and *wearable technology*	2021.01	Internet of Things as a platform to connect people and devices, for example, smart wearables and wearable biosensors [68] Wearable textiles [69] Wearables for sleep monitoring [70] Use of wearables during COVID-19 [71], for treating cancer [72], and for managing alcohol use disorder [73]

^a^The mean year of publication for each keyword was determined by averaging the publication dates of all documents associated with that keyword. To explore eHealth applications and other technology keywords interactively, use overlay links provided in Multimedia Appendix 1.

^b^mHealth: mobile health.

#### eHealth Stakeholders and Research Analysis Levels

We systematically categorized eHealth stakeholder groups and identified highly cited publications, including articles and reviews, that exemplify research involving these stakeholders. As demonstrated in Table 4, the studies we reviewed encompassed participants from all age groups, covering the entire lifespan [53,54,58,67,68,74-116].

**Table 4:** Highly cited eHealth papers by stakeholder groups.

User groups and keywords		Topical areas of highly cited papers listed in chronological order
**Age-based eHealth technology user groups**		
	*Young children* or *children*	eHealth literacy among parents of children with special care needs [74] eHealth solutions for pediatric asthma control [75] A review of digital mental health (computer-assisted therapy, smartphone apps, and wearable technologies) [76]
	*Adolescents*	Use of technology to find health information [77] A review of health literacy and health behaviors [78]
	*College/university students* or *nursing students*	Internet use to retrieve health information [79] A review of digital mental health interventions for depression, anxiety, psychological distress, and stress [80]
	*Young adults* and *adults*	Quality of mHealth^a^ apps [81] A review proposing a conceptual framework for engagement with digital behavior change interventions [82] Perceptions of video visits with established primary care clinicians [83]
	*Older adults, elderly people,* or *aged*	A review of aging in place [84] Health information–seeking behaviors [85] Reviews of smart homes (home health monitoring) [86] and physical activity interventions [87] A call for inclusive web-based and offline solutions to mitigate risks of COVID-19 [88] Reviews of telerehabilitation in physical therapy [89], barriers and facilitators to using eHealth technologies [90], and eHealth literacy as a mediator—how health-related information changes health-related behaviors [91]
**Other eHealth technology user groups**		
	*Patients*, *cancer patients*, or *cancer survivors*	Reviews of mHealth [92] and technology for patients with Parkinson disease [93] Recruitment into digital health interventions [94] Reviews of health literacy [95] and factors influencing eHealth intervention outcomes [96] Fog-driven internet of things (mHealth, assisted living, e-medicine, implants, and early warning systems) [68] Reviews of the internet of things and cloud computing [97], home consultation systems [98], digital solutions at the beginning of the COVID-19 pandemic [99], and telehealth use during COVID-19 [58] A web-based eHealth system for self-reporting symptoms during cancer treatment [53] A review of mHealth apps for prevention or management of noncommunicable diseases [54]
	*Family/informal caregivers* and *carers parents or mothers*	Internet-based technology for supporting self-care in primary care [100] A review of eHealth for cancer [101]
	*Healthcare professionals, physicians, nurses* or *doctors* and *health organizations*	A review of health care professionals’ role in eHealth implementation [102] Trust in health information from specialist physicians and dentists [103] Smart hospitals [104] EHR^b^ usability and nurses’ informatics competence [105] A review of web-based health misinformation [106] Medical data exchange through edge computing and blockchain [107]
	*Communities,* c*ountries,* or *societies*	Patients’ web-based communities [108] Societal impacts of internet of things [109], digital inequalities [110], COVID-19 infodemic [111], and digital literacy [112] Fog-driven internet of things architecture (population monitoring in smart cities) [68] Adoption of mHealth services in Bangladesh and low-income countries [67] Poor engagement with digital health by some communities [113] Health technologies for the internet of things in a 5G-enabled, fully connected society [114] A review of how the COVID-19 pandemic accelerated the digital transition across society [99] Desire for telemedicine in low-income countries [115] Health equity in digital health systems—a review of country-specific policies [116]

^a^mHealth: mobile health.

^b^EHR: electronic health record.

Our corpora offered evidence of eHealth research at the individual, community, country, and societal levels. At the individual level, researchers studied health consumers’ and health care professionals’ participation in telemedicine, mHealth, and web-based interventions. There was a strong focus on interventions to meet health needs in the areas of mental health, wellness, and chronic and infectious diseases, with an emphasis on outcomes such as self-management and self-care, prevention, behavior change, adherence, self-efficacy and motivation, health risk reduction, and mortality. Individual-level eHealth studies were not limited to interventions; they also included a large body of health literacy research, as well as descriptive studies of internet and social media use by individual health information seekers. At the organizational or health system level (refer to cluster 2 in Figure 2), researchers studied eHealth and medical records, patient portals, and health information and clinical decision support systems.

Several small nodes in our maps referred to communities, notably, *community* in Figure 3; *community, communities, and community-based participatory research* in Figure 2; and *community health* in Figure 6. In their abstracts, scholars discussed interventions for communities, aiming to produce community-level outcomes, for example, to promote knowledge exchange among geographically dispersed health care professionals or between laypeople and health care professionals (eg, [117]). In addition, eHealth scholars studied disease-specific web-based communities and conducted community-based participatory research to build a variety of eHealth tools for caregivers in support of their emotional, belongingness, and help-seeking needs.

The country and societal or global level was represented by 2 sets of studies. The first set included scholarship on new and emerging technologies with potential impact on all levels of eHealth, including the societal level: internet of things, cloud computing, blockchain, AI, etc. At the highest level, these technologies can be applied, for example for disease surveillance, secure data sharing, or population health predictions. It also included research about technology standardization, policy, ethics, and governance. Given the regional and global efforts to strategically allocate resources for health technologies, such as WHO’s Global Initiative on Digital Health [118], we expect an increase of relevant publications at the societal or global level, which may be indexed as “digital health” rather than “eHealth.” The second set of studies encompassed prepandemic and pandemic publications of health information available through global social media and the internet, with a focus on the quality and use of health information from electronic sources.

Within a proposed eHealth domain titled “health in our hands,” social media has been conceptualized as an “interacting for health” technology [7]. Although social media keywords appeared in our maps, node sizes were unexpectedly small, considering worldwide use of social media platforms [119] and health researchers’ interest in harnessing their power for health communication campaigns [120,121]. We likely failed to capture many relevant social media studies that did not mention eHealth in their titles, keywords, or abstracts. At the same time, an eHealth keyword is unlikely to be assigned to studies on problematic use of social media, for example, when social media contributes to adolescents’ poor mental health [122]. Underrepresentation of social media publications within eHealth is consistent with a claim that there may be a “dominant pathogenesis paradigm” in social media research [123], which manifests itself in the plethora of addiction scales and other measures of mental illness, physical inactivity, or poor sleep used by scholars in this domain [123]. Bringing social media under the eHealth (or digital health) umbrella will extend our knowledge of interventions that make use of support groups, virtual coaching, health education campaigns, etc. An alternative paradigm of “interacting for health” could stimulate experimental, longitudinal, and multilevel research conducted on social media platforms and websites with social media functionality. At the health system or societal level unit of analysis social media can be conceptualized as an eHealth technology in support of health policy, for example, for gathering digital publics’ input on health services and systems [124] or gauging public reactions to health policy issues, health systems, and organizations.

#### A Conceptual Model of eHealth

To the best of our best knowledge, this review represents one of the most comprehensive attempts to date to systematize research into eHealth by creating typologies of stakeholders, applications, and supporting technologies. Our findings align with earlier bibliometric studies of eHealth and digital health. We confirmed previously identified research directions, such as eHealth literacy [28,29], self-management [29], physical activity [28], mental health [28], COVID-19 [29], and technological infrastructures that support eHealth applications [25]. Consistent with our findings, a 2023 bibliometric review of internet of things research incorporating the search term “eHealth” demonstrated scholarship on blockchain applications, 5G networks, data analytics, and AI technologies [25]. We also replicated the finding that the term “digital health” was more recent than “eHealth” [29] and corroborated an earlier report regarding the pivotal role of JMIR journals within the field of eHealth [28]. In 2021, bibliometricians highlighted the role of social sciences, especially psychology, in eHealth scholarship [30]. We extended the findings of their study, which focused exclusively on eHealth articles in WoS’ social sciences research area, by demonstrating interconnections between psychology and other disciplines and depicting specific branches of psychology. According to our concept map, the highest number of studies were tagged as social psychology, followed by clinical, applied, developmental, cognitive, and health psychology. Despite eHealth applications’ emphasis on patient–health care professional communication, the field of communication was represented by small nodes in our concept map. Other social sciences—psychology, education, business, and economics—were more salient, which is another similarity between our study and an earlier bibliometric analysis of multidisciplinary contributions [30].

Building on the main points of the preceding discussion sections, Textbox 1 presents a conceptual model of eHealth scholarship. It consists of 5 building blocks that delineate the domain of eHealth as it has been explored over the past 25 years. The 5 blocks are stakeholders, applications, supporting infrastructures, analytics, and outcomes. Empirically derived categories depict the composition of each constituent building block.

**A bibliometrically derived conceptual model of eHealth stakeholders, technologies, and outcomes explored by eHealth scholars, from 2000 to 2024.**

**Designed for eHealth stakeholders**

- Children, adolescents, adults, and older adults
- Patients, family, and caregivers
- Health care professionals, organizations, and systems
- Communities, countries, and societies

**eHealth applications**

- Education and informatics: *e-learning* and *consumer health informatics*
- Telehealth: *telemedicine*, *telemonitoring*, and *telerehabilitation*
- Health record management: *electronic health records*, *patient portals*, and *personal health records*
- Mobile health (mHealth): *mobile apps* and *health apps*
- Wearables: *wearable technologies*

**And their supporting infrastructures**

- Networks, data exchange, computing technologies, hardware, information systems, and platforms
- Methods for managing data security and privacy

**With the help of health analytics**

- Artificial intelligence, algorithms, big data, data mining, deep learning, and machine learning

**Aim to achieve desired outcomes, such as:**

- Advanced public health, prevention, health literacy, and health education;
- Improved diagnosis, treatment, rehabilitation, quality of life, and well-being;
- Engagement, participation, and person-centeredness;
- Enhanced health system operations and interoperability.

#### How eHealth Research Developed Over Time

The earliest eHealth scholarship was rooted in computer and web technologies used for patient–health care professional communication and treatment of specific health conditions, as well as in telecare and medical informatics. In contrast, the most recent eHealth scholarship was represented by articles and reviews dedicated to the COVID-19 pandemic and a variety of newer technologies, such as AI, wearables, digital health, blockchain, and the internet of things. In our keyword maps, COVID-19 had strong links to telemedicine, expedited by the recent COVID-19 pandemic [61], and digital health. We also observed small nodes with recent research dedicated to cyberchondria and eHealth applications to promote mindfulness and treat urinary incontinence.

The keyword e-Health, as compared to eHealth, consistently indexed older articles and reviews, suggesting a shift toward terminology standardization. We recommend that library database managers and future authors consistently index their studies with the keyword eHealth to avoid problems in retrieving e-Health publications.

Another likely terminology shift is toward *digital health*, adopted in many WHO documents and defined as “the systematic application of information and communications technologies, computer science, and data to support informed decision-making by individuals, the health workforce, and health systems, to strengthen resilience to disease and improve health and wellness” [125]. Digital health has gained popularity as an umbrella term alternative to eHealth, according to our analyses. This finding confirmed nearly decade-long concerns documented by Shaw et al [7] in their interviews with eHealth researchers, educators, practitioners, and policy makers. One of their informants stated the following:

You know eHealth is really old fashioned? Nobody talks about eHealth anymore. Electronic health—everything’s electronic! The devices, everything! We’ re talking about digital health, digitizing health, not eHealth. [7]

P3

As more studies are indexed with digital health, the use of an eHealth keyword may decline. We recommend that future bibliometricians query both search terms to achieve historic depth of their corpora for tracking this research field’s evolution.

#### The Multidisciplinary Nature of eHealth

We observed multidisciplinarity in both inputs and outputs of eHealth science, that is, in foundational literature presented as the names of cited journals and disciplinary tags assigned to eHealth publications by OpenAlex. Some of the journals were technology oriented (*Journal of Medical Internet Research)*, telemedicine journals, or journals about sensors); however, most cited journals were not specific to health technology, suggesting broad support for eHealth application studies from a variety of medical fields. Psychology, psychiatry, public health, and preventive medicine journals were prominent in our source co-occurrence map. Other journals were specific to age groups, ranging from pediatric to gerontological sources, which suggests that eHealth draws upon literature concerned with health and wellness across the human lifespan. According to our OpenAlex concept map and its mean publication year overlay, the eHealth scholarship originated as computer science and engineering research in support of medicine, nursing, and public health, with ongoing contributions by eHealth literacy scholars. The core interest of eHealth—technological innovations and interventions—was supported by disciplines concerned with policy, law, and economy. The eHealth research domain, therefore, extends well beyond medical and health technologies, encompassing a wide range of other disciplines.

These findings have implications for policy makers, practitioners, those who shape research priorities, and educators. First, it may be challenging to identify individuals with expertise in both health and technology [126]; therefore, policy development panels and teams involved in eHealth research or implementation may need to include both health specialists and technology experts. Our visualization of disciplines relevant to the eHealth domain supports resource allocation beyond the intersection of technology and health, extending to a broader range of fields, such as, health literacy and social sciences. Effective policy and regulatory framework development for eHealth must account for the technological, clinical, legal, ethical, economic, and societal implications of eHealth solutions.

Second, funding of studies conducted by multidisciplinary research teams should be prioritized by research funders to bring together researchers and relevant stakeholder groups, such as clinicians, public health officials, health care leaders, patients and health advocacy groups, technologists or industry representatives, policy makers, and community members. Within each eHealth research subdomain, defined by our map clusters, engagement of relevant stakeholder groups and user panels (eg, those listed in Multimedia Appendix 1) may help to ensure that future research addresses real-world needs and concerns, while also enhancing the relevance of research findings and their translation into practice.

Third, educators can use our visual representations as teaching aids. Big-picture perspectives communicated in our maps offer a holistic view of the eHealth research domain. For example, individuals involved in health care management, public health, and digital health education may use maps to introduce their students to the multidisciplinary field of eHealth. They can position their respective disciplines within the network of other contributing fields to emphasize the benefits of multidisciplinarity, collaboration, and broad knowledge acquisition. Educators may also encourage students to research concepts that appear within the keyword and concept maps to find inspiration outside of the core literature they are typically exposed to within their academic programs. Finally, the maps may also serve as an exploratory starting point for novice researchers who are interested in eHealth or digital health and need to narrow down their research focus.

### Limitations

One of the study limitations is the exclusion of e-Health articles from the OpenAlex search query to avoid potential issues with the hyphenated search term. The literature of interest is undoubtedly broader than the 10,022 articles we mapped. Moreover, our study addressed high-level patterns in metadata, limiting visualizations to keywords and concepts that met preset occurrence thresholds, specifically keywords used in no less than 10 studies and concepts tagging ≥20 OpenAlex articles. Less frequently used keywords and concepts were excluded, some of which were likely very useful in understanding eHealth.

Our study revealed a recent trend to index eHealth studies with a digital health keyword. Mapping the literature using search queries that include both “eHealth” and “digital health” would provide the most comprehensive and up-to-date depiction of the research domain. Therefore, future bibliometric research should incorporate both terms.

While this bibliometric study did not offer a summary of evidence that can be expected from traditional systematic reviews, for example, on integrating eHealth solutions into practice, it provided a high-level overview of past research, pertinent terminology, and interlinked research directions, which were categorized and synthesized to develop a conceptual model of eHealth scholarship.

### Practical Implications and Suggestions for Future Research

The implications of our findings are presented in Textbox 2, and recommendations for future studies are presented in Textbox 3. The implications are organized into 3 groups: practical implications of our conceptual model and maps, availability of numerous and recent reviews in many eHealth research areas, and multidisciplinary contributions to eHealth. For future researchers, we offer recommendations on research directions, as well as guidance on study design and publication.

**Study implications.**

**The value of our conceptual model and interactive bibliometric networks**

- Practitioners, educators, and novice researchers can use this study’s maps, terminology, typologies, and conceptual models to gain a comprehensive understanding of eHealth, expanding the boundaries of their academic or practice fields.
- Mapped concepts and keywords align with the established terminology, aiding researchers and practitioners in the selection of eHealth outcomes and the identification of measurement instruments.
- Mean publication recency overlays can inform funders’ decisions to stimulate research on emerging topics that are currently represented by a small number of studies (eg, mindfulness, urinary incontinence, and cyberchondria).

**Availability of synthesized evidence from eHealth reviews**

- To keep pace with new research developments, researchers and practitioners may turn to recent eHealth systematic reviews, especially those synthesizing evidence on interventions, common eHealth applications, and feasibility studies.
- The keyword map for reviews (Figure 3) can be used to develop queries for identifying reviews of interest.

**Significance of multidisciplinary contributions of eHealth**

- Informed policy and regulatory framework development should consider the technological, clinical, legal, ethical, economic, and other societal implications of eHealth solutions.
- Funders should extend support to scholars and practitioners from diverse disciplines identified in our study. They should also consider funding research from underrepresented disciplines, such as communication.
- Funders should also support research engagement of relevant stakeholder groups and user panels, including the groups we identified.

**Suggestions for future research.**

**Research directions**

- Our maps can serve as an exploratory starting point for novice researchers who are interested in eHealth and need to narrow down their research focus.
- Social media research under the eHealth umbrella (eg, media support groups, virtual coaching, health education campaigns, and other social media “interacting for health” interventions) will promote the “social media for health” paradigm, which is distinctly different from the dominant pathogenesis paradigm.

**Design considerations**

- The following eHealth topical areas with abundant literature and relatively few reviews may be considered by systematic review authors: eHealth literacy; data privacy and security; eHealth technology adoption (barriers, usability, and user acceptance); and mental health topics of social support, self-efficacy, and eHealth applications of cognitive behavioral therapy.
- Researchers should consider multiple units of analysis when designing studies and identifying outcome variables.
- Bibliometricians aiming for historical depth in their corpora should include search terms such as e-Health, eHealth, and possibly digital health to ensure.

**Guidance on publishing new studies**

- Researchers should index their studies with a keyword eHealth rather than e-Health for consistent retrieval.
- Researchers can identify potential journals for submitting their studies by using the interactive cocitation network of sources we developed.

### Conclusions

The multidisciplinary field of study at the crossroads of health and technology is widely recognized as eHealth. Over the past 25 years, researchers studied a broad range of established and emerging technologies—educational and informatics tools, telehealth services, health record management, mHealth, and wearables—in support of consumer-oriented solutions for patient monitoring, diagnosis, treatment, rehabilitation, and patient– health care professional communication. Beyond health care services, the field of eHealth offers a large body of literature on health literacy, disease prevention, and wellness. Conducted at the individual, health system, community, and society levels, eHealth research continues to develop by incorporating new technologies, responding to health emergencies, and addressing the needs of diverse stakeholders.

## Appendix

Multimedia Appendix 1 Links to interactive maps, map overlays, and publication impact.

## Abbreviations

AI: artificial intelligence
LIWC: linguistic inquiry and word count
PRISMA: Preferred Reporting Items for Systematic Reviews and Meta-Analyses
RQ: research question
WHO: World Health Organization
WoS: Web of Science

## Data Availability

The datasets generated or analyzed during this study are available from the corresponding author on reasonable request. Map files can be downloaded from the map URLs provided in Multimedia Appendix 1.

## References

[1] eHealth: health topics. World Health Organization.

[2] Mann DM, Chen J, Chunara R, Testa PA, Nov O (2020). COVID-19 transforms health care through telemedicine: evidence from the field. J Am Med Inform Assoc.

[3] Alonso SG, Marques G, Barrachina I, Garcia-Zapirain B, Arambarri J, Salvador JC, de la Torre Díez I (2021). Telemedicine and e-Health research solutions in literature for combatting COVID-19: a systematic review. Health Technol (Berl).

[4] Rowland SP, Fitzgerald JE, Holme T, Powell J, McGregor A (2020). What is the clinical value of mHealth for patients?. NPJ Digit Med.

[5] Rajpurkar P, Chen E, Banerjee O, Topol EJ (2022). AI in health and medicine. Nat Med.

[6] Barello S, Triberti S, Graffigna G, Libreri C, Serino S, Hibbard J, Riva G (2015). eHealth for patient engagement: a systematic review. Front Psychol.

[7] Shaw T, McGregor D, Brunner M, Keep M, Janssen A, Barnet S (2017). What is eHealth (6)? Development of a conceptual model for eHealth: qualitative study with key informants. J Med Internet Res.

[8] Donthu N, Kumar S, Mukherjee D, Pandey N, Lim WM (2021). How to conduct a bibliometric analysis: an overview and guidelines. J Bus Res.

[9] Muller AM, Maher CA, Vandelanotte C, Hingle M, Middelweerd A, Lopez ML, DeSmet A, Short CE, Nathan N, Hutchesson MJ, Poppe L, Woods CB, Williams SL, Wark PA (2018). Physical activity, sedentary behavior, and diet-related eHealth and mHealth research: bibliometric analysis. J Med Internet Res.

[10] Fang T, Cao H, Wang Y, Gong Y, Wang Z (2023). Global scientific trends on healthy eating from 2002 to 2021: a bibliometric and visualized analysis. Nutrients.

[11] Železnik U, Kokol P, Starc J, Železnik D, Završnik J, Vošner HB (2023). Research trends in motivation and weight loss: a bibliometric-based review. Healthcare (Basel).

[12] Palozzi G, Antonucci G (2022). Mobile-health based physical activities co-production policies towards cardiovascular diseases prevention: findings from a mixed-method systematic review. BMC Health Serv Res.

[13] Johansson M, Romero D, Jakobson M, Heinemans N, Lindner P (2024). Digital interventions targeting excessive substance use and substance use disorders: a comprehensive and systematic scoping review and bibliometric analysis. Front Psychiatry.

[14] Ellis LA, Meulenbroeks I, Churruca K, Pomare C, Hatem S, Harrison R, Zurynski Y, Braithwaite J (2021). The application of e-Mental health in response to COVID-19: scoping review and bibliometric analysis. JMIR Ment Health.

[15] Taj F, Klein MC, van Halteren A (2019). Digital health behavior change technology: bibliometric and scoping review of two decades of research. JMIR Mhealth Uhealth.

[16] Wu Y, Wang X, Zhou M, Huang Z, Liu L, Cong L (2024). Application of eHealth tools in anticoagulation management after cardiac valve replacement: scoping review coupled with bibliometric analysis. JMIR Mhealth Uhealth.

[17] Kokol P, Saranto K, Blažun Vošner H (2018). eHealth and health informatics competences: a systemic analysis of literature production based on bibliometrics. Kybernetes.

[18] Wang C, Wu X, Qi H (2021). A comprehensive analysis of e-Health literacy research focuses and trends. Healthcare (Basel).

[19] Calvo F, Carbonell X, Johnsen S (2019). Information and communication technologies, e-Health and homelessness: a bibliometric review. Cogent Psychol.

[20] Tapera R, Singh Y (2021). A bibliometric analysis of medical informatics and telemedicine in sub-Saharan Africa and BRICS nations. J Public Health Res.

[21] Lwin HN, Punnakitikashem P, Thananusak T (2023). e-Health research in southeast Asia: a bibliometric review. Sustainability.

[22] Puliga G, Nasullaev A, Bono F, Gutiérrez E, Strozzi F (2020). Ambient assisted living and European funds: a bibliometric approach. Inf Technol People.

[23] Cobelli N, Blasioli E (2023). To be or not to be digital? A bibliometric analysis of adoption of eHealth services. TQM J.

[24] Sahoo S, Sahoo J, Kumar S, Lim WM, Ameen N (2023). Distance is no longer a barrier to healthcare services: current state and future trends of telehealth research. Internet Res.

[25] Rejeb A, Rejeb K, Treiblmaier H, Appolloni A, Alghamdi S, Alhasawi Y, Iranmanesh M (2023). The Internet of Things (IoT) in healthcare: taking stock and moving forward. Internet Things.

[26] Palozzi G, Schettini I, Chirico A (2020). Enhancing the sustainable goal of access to healthcare: findings from a literature review on telemedicine employment in rural areas. Sustainability.

[27] Shaikh AK, Alhashmi SM, Khalique N, Khedr AM, Raahemifar K, Bukhari S (2023). Bibliometric analysis on the adoption of artificial intelligence applications in the e-health sector. Digit Health.

[28] Tian H, Chen J (2022). A bibliometric analysis on global eHealth. Digit Health.

[29] Sikandar H, Abbas AF, Khan N, Qureshi MI (2022). Digital technologies in healthcare: a systematic review and bibliometric analysis. Int J online Biomed Eng.

[30] Uribe-Toril J, Ruiz-Real JL, Nievas-Soriano BJ (2021). A study of eHealth from the perspective of social sciences. Healthcare (Basel).

[31] van Eck NJ, Waltman L VOSviewer manual 1.6.20. University of Leiden.

[32] (2024). Concepts. OpenAlex Technical Documentation.

[33] van Eck NJ, Waltman L (2017). Citation-based clustering of publications using CitNetExplorer and VOSviewer. Scientometrics.

[34] Waltman L, van Eck NJ, Noyons EC (2010). A unified approach to mapping and clustering of bibliometric networks. J Informetr.

[35] A keyword co-occurrence network for 5000 eHealth articles: a cluster map. Keywords that occur ≥10 times were mapped. VOSviewer.

[36] A keyword co-occurrence network for 1885 eHealth reviews: a cluster map. Keywords that occur ≥10 times were mapped. VOSviewer.

[37] A keyword co-occurrence network for 5,000 eHealth articles, interactive map. VOSviewer.

[38] Rockers PC, Røttingen JA, Shemilt I, Tugwell P, Bärnighausen T (2015). Inclusion of quasi-experimental studies in systematic reviews of health systems research. Health Policy.

[39] Publication recency overlays to maps in Figures 1 and 2: keywords indexing articles (top) and reviews (bottom) [articles]. VOSviewer.

[40] Publication recency overlays to maps in Figures 1 and 2: keywords indexing articles (top) and reviews (bottom) [reviews]. VOSviewer.

[41] A cocitation network of sources for 1885 eHealth reviews: a cluster map. VOSviewer.

[42] A concept co-occurrence network for 10,022 eHealth articles from OpenAlex: a cluster map. VOSviewer.

[43] Mean year for all articles represented by a concept. VOSviewer.

[44] eHealth technology or related concept. VOSviewer.

[45] eHealth objective (a concept related to desired outcomes or goals). VOSviewer.

[46] Health issues or field (broadly defined concepts related to health and health disciplines, including illness, wellness, and mental health). VOSviewer.

[47] Illness (a concept specific to diseases and health conditions). VOSviewer.

[48] Wellness (a concept specific to health promotion and maintenance). VOSviewer.

[49] Mental health (a concept related to cognitive, behavioral, and emotional well-being). VOSviewer.

[50] Risk (a concept related to risk in technology or health domains). VOSviewer.

[51] Economics and business (economics and business related concept or field). VOSviewer.

[52] Essential science indicators - highly cited papers. Clarivate.

[53] Absolom K, Warrington L, Hudson E, Hewison J, Morris C, Holch P, Carter R, Gibson A, Holmes M, Clayton B, Rogers Z, McParland L, Conner M, Glidewell L, Woroncow B, Dawkins B, Dickinson S, Hulme C, Brown J, Velikova G (2021). Phase III randomized controlled trial of eRAPID: eHealth intervention during chemotherapy. JCO.

[54] Jakob R, Harperink S, Rudolf AM, Fleisch E, Haug S, Mair JL, Salamanca-Sanabria A, Kowatsch T (2022). Factors influencing adherence to mHealth apps for prevention or management of noncommunicable diseases: systematic review. J Med Internet Res.

[55] Keselman A, Smith CA, Murcko AC, Kaufman DR (2019). Evaluating the quality of health information in a changing digital ecosystem. J Med Internet Res.

[56] Ouyang W, Xie W, Xin Z, He H, Wen T, Peng X, Dai P, Yuan Y, Liu F, Chen Y, Luo A (2021). Evolutionary overview of consumer health informatics: bibliometric study on the web of science from 1999 to 2019. J Med Internet Res.

[57] Ekeland AG, Bowes A, Flottorp S (2010). Effectiveness of telemedicine: a systematic review of reviews. Int J Med Inform.

[58] Garfan S, Alamoodi AH, Zaidan BB, Al-Zobbi M, Hamid RA, Alwan JK, Ahmaro IY, Khalid ET, Jumaah F, Albahri O, Zaidan A, Albahri A, Al-Qaysi ZT, Ahmed M, Shuwandy ML, Salih MM, Zughoul O, Mohammed K, Momani F (2021). Telehealth utilization during the COVID-19 pandemic: a systematic review. Comput Biol Med.

[59] Haleem A, Javaid M, Singh RP, Suman R (2021). Telemedicine for healthcare: capabilities, features, barriers, and applications. Sens Int.

[60] Riley RD, Ensor J, Snell KI, Debray TP, Altman DG, Moons KG, Collins GS (2016). External validation of clinical prediction models using big datasets from e-health records or IPD meta-analysis: opportunities and challenges. BMJ.

[61] Roehrs A, da Costa CA, da Rosa Righi R (2017). OmniPHR: a distributed architecture model to integrate personal health records. J Biomed Inform.

[62] Zhang A, Lin X (2018). Towards secure and privacy-preserving data sharing in e-Health systems via consortium blockchain. J Med Syst.

[63] Antonio MG, Petrovskaya O, Lau F (2019). Is research on patient portals attuned to health equity? A scoping review. J Am Med Inform Assoc.

[64] Brands MR, Gouw SC, Beestrum M, Cronin RM, Fijnvandraat K, Badawy SM (2022). Patient-centered digital health records and their effects on health outcomes: systematic review. J Med Internet Res.

[65] Bol N, Helberger N, Weert JC (2018). Differences in mobile health app use: a source of new digital inequalities?. Inf Soc.

[66] Rajak M, Shaw K (2019). Evaluation and selection of mobile health (mHealth) applications using AHP and fuzzy TOPSIS. Technol Soc.

[67] Alam MZ, Hoque MR, Hu W, Barua Z (2020). Factors influencing the adoption of mHealth services in a developing country: a patient-centric study. Int J Inf Manage.

[68] Farahani B, Firouzi F, Chang V, Badaroglu M, Constant N, Mankodiya K (2018). Towards fog-driven IoT eHealth: promises and challenges of IoT in medicine and healthcare. Future Gener Comput Syst.

[69] Acar G, Ozturk O, Golparvar AJ, Elboshra TA, Böhringer K, Yapici MK (2019). Wearable and flexible textile electrodes for biopotential signal monitoring: a review. Electronics.

[70] Guillodo E, Lemey C, Simonnet M, Walter M, Baca-García E, Masetti V, Moga S, Larsen M, Ropars J, Berrouiguet S, HUGOPSY Network (2020). Clinical applications of mobile health wearable-based sleep monitoring: systematic review. JMIR Mhealth Uhealth.

[71] Channa A, Popescu N, Skibinska J, Burget R (2021). The rise of wearable devices during the COVID-19 pandemic: a systematic review. Sensors (Basel).

[72] Huang Y, Upadhyay U, Dhar E, Kuo LJ, Syed-Abdul S (2022). A scoping review to assess adherence to and clinical outcomes of wearable devices in the cancer population. Cancers (Basel).

[73] Kruse CS, Betancourt JA, Madrid S, Lindsey CW, Wall V (2022). Leveraging mHealth and wearable sensors to manage alcohol use disorders: a systematic literature review. Healthcare (Basel).

[74] Knapp C, Madden V, Wang H, Sloyer P, Shenkman E (2011). Internet use and eHealth literacy of low-income parents whose children have special health care needs. J Med Internet Res.

[75] Gustafson D, Wise M, Bhattacharya A, Pulvermacher A, Shanovich K, Phillips B, Lehman E, Chinchilli V, Hawkins R, Kim J (2012). The effects of combining web-based eHealth with telephone nurse case management for pediatric asthma control: a randomized controlled trial. J Med Internet Res.

[76] Hollis C, Falconer CJ, Martin JL, Whittington C, Stockton S, Glazebrook C, Davies EB (2017). Annual research review: digital health interventions for children and young people with mental health problems - a systematic and meta-review. J Child Psychol Psychiatry.

[77] Skinner H, Biscope S, Poland B, Goldberg E (2003). How adolescents use technology for health information: implications for health professionals from focus group studies. J Med Internet Res.

[78] Fleary SA, Joseph P, Pappagianopoulos JE (2018). Adolescent health literacy and health behaviors: a systematic review. J Adolesc.

[79] Escoffery C, Miner KR, Adame DD, Butler S, McCormick L, Mendell E (2005). Internet use for health information among college students. J Am Coll Health.

[80] Davies EB, Morriss RK, Glazebrook C (2014). Computer-delivered and web-based interventions to improve depression, anxiety, and psychological well-being of university students: a systematic review and meta-analysis. J Med Internet Res.

[81] Stoyanov SR, Hides L, Kavanagh DJ, Wilson H (2016). Development and validation of the user version of the mobile application rating scale (uMARS). JMIR Mhealth Uhealth.

[82] Perski O, Blandford A, West R, Michie S (2017). Conceptualising engagement with digital behaviour change interventions: a systematic review using principles from critical interpretive synthesis. Transl Behav Med.

[83] Powell RE, Henstenburg JM, Cooper G, Hollander JE, Rising KL (2017). Patient perceptions of telehealth primary care video visits. Ann Fam Med.

[84] Peek ST, Wouters EJ, van Hoof J, Luijkx KG, Boeije HR, Vrijhoef HJ (2014). Factors influencing acceptance of technology for aging in place: a systematic review. Int J Med Inform.

[85] Tennant B, Stellefson M, Dodd V, Chaney B, Chaney D, Paige S, Alber J (2015). eHealth literacy and web 2.0 health information seeking behaviors among baby boomers and older adults. J Med Internet Res.

[86] Liu L, Stroulia E, Nikolaidis I, Miguel-Cruz A, Rios Rincon A (2016). Smart homes and home health monitoring technologies for older adults: a systematic review. Int J Med Inform.

[87] Muellmann S, Forberger S, Möllers T, Bröring E, Zeeb H, Pischke CR (2018). Effectiveness of eHealth interventions for the promotion of physical activity in older adults: a systematic review. Prev Med.

[88] Xie B, Charness N, Fingerman K, Kaye J, Kim MT, Khurshid A (2020). When going digital becomes a necessity: ensuring older adults' needs for information, services, and social inclusion during COVID-19. J Aging Soc Policy.

[89] Seron P, Oliveros MJ, Gutierrez-Arias R, Fuentes-Aspe R, Torres-Castro RC, Merino-Osorio C, Nahuelhual P, Inostroza J, Jalil Y, Solano R, Marzuca-Nassr GN, Aguilera-Eguía R, Lavados-Romo P, Soto-Rodríguez FJ, Sabelle C, Villarroel-Silva G, Gomolán P, Huaiquilaf S, Sanchez P (2021). Effectiveness of telerehabilitation in physical therapy: a rapid overview. Phys Ther.

[90] Wilson J, Heinsch M, Betts D, Booth D, Kay-Lambkin F (2021). Barriers and facilitators to the use of e-health by older adults: a scoping review. BMC Public Health.

[91] Kim K, Shin S, Kim S, Lee E (2023). The relation between eHealth literacy and health-related behaviors: systematic review and meta-analysis. J Med Internet Res.

[92] Silva BM, Rodrigues JJ, de la Torre Díez I, López-Coronado M, Saleem K (2015). Mobile-health: a review of current state in 2015. J Biomed Inform.

[93] Espay AJ, Bonato P, Nahab FB, Maetzler W, Dean JM, Klucken J, Eskofier BM, Merola A, Horak F, Lang AE, Reilmann R, Giuffrida J, Nieuwboer A, Horne M, Little MA, Litvan I, Simuni T, Dorsey ER, Burack MA, Kubota K, Kamondi A, Godinho C, Daneault J, Mitsi G, Krinke L, Hausdorff JM, Bloem BR, Papapetropoulos S, Movement Disorders Society Task Force on Technology (2016). Technology in Parkinson's disease: challenges and opportunities. Mov Disord.

[94] O'Connor S, Hanlon P, O'Donnell CA, Garcia S, Glanville J, Mair FS (2016). Understanding factors affecting patient and public engagement and recruitment to digital health interventions: a systematic review of qualitative studies. BMC Med Inform Decis Mak.

[95] Kim H, Xie B (2017). Health literacy in the eHealth era: a systematic review of the literature. Patient Educ Couns.

[96] Granja C, Janssen W, Johansen MA (2018). Factors determining the success and failure of eHealth interventions: systematic review of the literature. J Med Internet Res.

[97] Dang LM, Piran MJ, Han D, Min K, Moon H (2019). A survey on internet of things and cloud computing for healthcare. Electronics.

[98] Almathami HK, Win KT, Vlahu-Gjorgievska E (2020). Barriers and facilitators that influence telemedicine-based, real-time, online consultation at patients' homes: systematic literature review. J Med Internet Res.

[99] Golinelli D, Boetto E, Carullo G, Nuzzolese AG, Landini MP, Fantini MP (2020). Adoption of digital technologies in health care during the COVID-19 pandemic: systematic review of early scientific literature. J Med Internet Res.

[100] Nijland N, van Gemert-Pijnen J, Boer H, Steehouder MF, Seydel ER (2008). Evaluation of internet-based technology for supporting self-care: problems encountered by patients and caregivers when using self-care applications. J Med Internet Res.

[101] Slev VN, Mistiaen P, Pasman HR, Verdonck-de Leeuw IM, van Uden-Kraan CF, Francke AL (2016). Effects of eHealth for patients and informal caregivers confronted with cancer: a meta-review. Int J Med Inform.

[102] Ross J, Stevenson F, Lau R, Murray E (2016). Factors that influence the implementation of e-health: a systematic review of systematic reviews (an update). Implement Sci.

[103] Chen X, Hay JL, Waters EA, Kiviniemi MT, Biddle C, Schofield E, Li Y, Kaphingst K, Orom H (2018). Health literacy and use and trust in health information. J Health Commun.

[104] Rahmani AM, Gia TN, Negash B, Anzanpour A, Azimi I, Jiang M, Liljeberg P (2018). Exploiting smart e-Health gateways at the edge of healthcare internet-of-things: a fog computing approach. Future Gener Comput Syst.

[105] Vehko T, Hyppönen H, Puttonen S, Kujala S, Ketola E, Tuukkanen J, Aalto A, Heponiemi T (2019). Experienced time pressure and stress: electronic health records usability and information technology competence play a role. BMC Med Inform Decis Mak.

[106] Swire-Thompson B, Lazer D (2020). Public health and online misinformation: challenges and recommendations. Annu Rev Public Health.

[107] Abdellatif AA, Samara L, Mohamed A, Erbad A, Chiasserini CF, Guizani M, O’Connor MD, Laughton J (2021). MEdge-chain: leveraging edge computing and blockchain for efficient medical data exchange. IEEE Internet Things J.

[108] Josefsson U (2005). Coping with illness online: the case of patients' online communities. Inf Soc.

[109] Islam SM, Daehan Kwak D, Humaun Kabir M, Hossain M, Kyung-Sup Kwak KS (2015). The internet of things for health care: a comprehensive survey. IEEE Access.

[110] Robinson L, Cotten SR, Ono H, Quan-Haase A, Mesch G, Chen W, Schulz J, Hale TM, Stern MJ (2015). Digital inequalities and why they matter. Inf Commun Soc.

[111] Pian W, Chi J, Ma F (2021). The causes, impacts and countermeasures of COVID-19 "infodemic": a systematic review using narrative synthesis. Inf Process Manag.

[112] Campanozzi LL, Gibelli F, Bailo P, Nittari G, Sirignano A, Ricci G (2023). The role of digital literacy in achieving health equity in the third millennium society: a literature review. Front Public Health.

[113] Crawford A, Serhal E (2020). Digital health equity and COVID-19: the innovation curve cannot reinforce the social gradient of health. J Med Internet Res.

[114] Qadri YA, Nauman A, Zikria YB, Vasilakos AV, Kim SW (2020). The future of healthcare internet of things: a survey of emerging technologies. IEEE Commun Surv Tutor.

[115] Kashani MH, Madanipour M, Nikravan M, Asghari P, Mahdipour E (2021). A systematic review of IoT in healthcare: applications, techniques, and trends. J Netw Comput Appl.

[116] van Kessel R, Hrzic R, O'Nuallain E, Weir E, Wong BL, Anderson M, Baron-Cohen S, Mossialos E (2022). Digital health paradox: international policy perspectives to address increased health inequalities for people living with disabilities. J Med Internet Res.

[117] Sims JM (2018). Communities of practice: telemedicine and online medical communities. Technol Forecast Soc Change.

[118] Global initiative on digital health. World Health Organization.

[119] Americans’ social media use. Pew Research Center.

[120] Rayward AT, Vandelanotte C, Corry K, Van Itallie A, Duncan MJ (2019). Impact of a social media campaign on reach, uptake, and engagement with a free web- and app-based physical activity intervention: the 10,000 steps Australia program. Int J Environ Res Public Health.

[121] Namkoong K, Nah S, Van Stee SK, Record R (2018). Social media campaign effects: moderating role of social capital in an anti-smoking campaign. Health Commun.

[122] (2024). Surgeon general: why I’m calling for a warning label on social media platforms. New York Times.

[123] Bekalu MA, Sato T, Viswanath K (2023). Conceptualizing and measuring social media use in health and well-being studies: systematic review. J Med Internet Res.

[124] Ivanitskaya LV, Erzikova EV (2025). Visualizing YouTube commenters' conceptions of the US health care system: semantic network analysis method for evidence-based policy making. JMIR Infodemiology.

[125] Classification of digital interventions, services and applications in health: a shared language to describe the uses of digital technology for health. 2nd edition. World Health Organization (WHO).

[126] Pfob A, Sidey-Gibbons C, Schuessler M, Lu S, Xu C, Dubsky P, Golatta M, Heil J (2021). Contrast of digital and health literacy between IT and health care specialists highlights the importance of multidisciplinary teams for digital health—a pilot study. JCO Clin Cancer Inform.

