# Supplementary material for: Multidisciplinary Contributions and Research Trends in eHealth Scholarship (2000-2024): Bibliometric Analysis
Source: J Med Internet Res. 2025 Jun 16;27:e60071. doi: 10.2196/60071 (PMC12209731; doi:10.2196/60071)
Supplement: Multimedia Appendix 1 [file jmir_v27i1e60071_app1.docx]

# Multimedia Appendix 1

Table of Contents

[Multimedia Appendix 1 1](#_Toc198567749)

[1. Interactive Links to Figures with Maps and Overlays 2](#_Toc198567750)

[Figure 2 2](#_Toc198567751)

[Figure 3 2](#_Toc198567752)

[Figure 5 3](#_Toc198567753)

[Figure 6 3](#_Toc198567754)

[Table 1 4](#_Toc198567755)

[2. Publication Impact: Mean Citations and Mean Normalized Citations 11](#_Toc198567756)

[Methods 11](#_Toc198567757)

[Results 12](#_Toc198567758)

[Publication Impact: Mean Normalized Citations for Web of Science Articles and Reviews 12](#_Toc198567759)

[Publication Impact: Mean Normalized Citations for eHealth Studies from OpenAlex 12](#_Toc198567760)

# 1. Interactive Links to Figures with Maps and Overlays

## Figure 2

A keyword co-occurrence network for 5000 eHealth articles: a cluster map, <https://tinyurl.com/2yr4vj6g>

Map overlays:

- Who: groups involved in eHealth, <https://tinyurl.com/2d7h2z87>
- What: conditions, needs, and care settings, <https://tinyurl.com/2bko7z7h>
- How: eHealth technologists and ideas, <https://tinyurl.com/2xhg93ha>
- eHealth umbrella terminology, <https://tinyurl.com/2xtjghnx>
- eHealth applications terminology, <https://tinyurl.com/23tv26lc>
- eHealth objectives terminology, <https://tinyurl.com/28ju6osl>
- Technology infrastructure terminology, <https://tinyurl.com/2dldhubv>
- Data security and privacy terminology, <https://tinyurl.com/2dep8o6e>
- Health analytics terminology, <https://tinyurl.com/2bx232h5>
- Publication recency (mean year for all studies represented by a keyword), <https://tinyurl.com/2dgeo5u2>
- Also, see links in section 2 of this document

## Figure 3

A keyword co-occurrence network for 1885 eHealth reviews: a cluster map, <https://tinyurl.com/28o3h7hs>

Map overlays:

- Publication recency (mean year for all reviews represented by a keyword), <https://tinyurl.com/24tkgc5z>
- Also, see links in section 2 of this document

## Figure 5

A cocitation network of sources for 1885 eHealth reviews: a cluster map, <https://tinyurl.com/274ugxye>. This map has no overlays.

## Figure 6

A concept co-occurrence network for 10,022 eHealth articles from OpenAlex: a cluster map, <https://tinyurl.com/23gsmjc4>

Map overlays:

- eHealth technology or related concept, <https://tinyurl.com/23gsmjc4>
- eHealth objective (a concept related to desired outcomes or goals), <https://tinyurl.com/2a834blf>
- eHealth objective (a concept related to desired outcomes or goals), <https://tinyurl.com/2a834blf>
- Health issues or field (a broadly defined concept related to health and health disciplines, including illness, wellness, and mental health), <https://tinyurl.com/2cqsn3yb>
- Illness (a concept specific to diseases and health conditions), <https://tinyurl.com/2ybh33ne>
- Wellness (a concept specific to health promotion and maintenance), <https://tinyurl.com/2avqly69>
- Mental health (a concept related to cognitive, behavioral, and emotional well-being), <https://tinyurl.com/23hcfw2n>
- Risk (a concept related to risk in technology or health domains), <https://tinyurl.com/27e7agsm>
- Economics and business related concept or field, <https://tinyurl.com/2ajltxvq>
- Publication recency (mean year for all studies represented by a concept), <https://tinyurl.com/27wsed82>
- Also, see links in section 2 of this document

## Table 1

Keywords indexing eHealth research articles, by cluster: who, what, and how of eHealth.

| Cluster number (color^a^) and name | Who: keywords^b^ that designate groups involved with eHealth | What: keywords^b^ about health conditions, needs, or care settings | How: eHealth technologies and technology-related keywords^b^ |
| --- | --- | --- | --- |
| 1 (Red): self-management and interventions for mental health, dementia, cancer, and pain | *children, people, adolescents, women, parents, adolescent, caregivers, survivors, youth, cancer survivors, cancer-patients, child, family, pediatrics, childhood, individuals, young-people, carers, families, family caregivers, parent, young-adults, caregiver, informal caregivers, mothers, young adult,* and *young-children* | *self-management, depression, cancer, quality-of-life, anxiety, mental health, social support, mental health, quality of life, stress, dementia, breast cancer, cognitive-behavioral therapy, oncology, chronic pain, breast-cancer, pain, chronic illness, disability, cognitive-behavior therapy, psychotherapy, fatigue, palliative care, mindfulness, distress, psychological distress, supportive care, schizophrenia, disorder, patient activation, alzheimers-disease, cognitive behavioral therapy, illness, osteoarthritis, hospital anxiety, sleep, substance use, functional assessment, glycemic control, health-related quality of life, help-seeking, anxiety disorders, cancer survivorship, depressive symptoms, low-back-pain, psychological treatments, symptom management, cancer care, insomnia, urinary incontinence, well-being, behavioral health, comorbidity, loneliness, person-centred care, postpartum depression,* and *self-management program* | *ehealth intervention, web-based intervention, e-learning, mobile apps, version, mobile app, e-mental health, web-based,* and *online intervention* |
| 2 (Green): telemedicine, telehealth, telecare, and technology acceptance | *patient, physicians, nurses, professionals, developing countries, older people, saudi arabia, patients, facilitators, communities, europe, physician, africa, australia, developing-countries, doctors, leadership, european union, healthcare professionals, middle-income countries,* and *stakeholders* | *access, primary health care, empowerment, patient empowerment, patient-centered care, patient participation, disease management, patient engagement, consultation, human factors, telepsychiatry, behavioral intention, information-seeking behavior, patient safety,* and *peer support* | *telemedicine, technology, telehealth, information-technology, usability, electronic health records, user acceptance, information systems, electronic health record, records, health informatics, technology acceptance model, telecare, ict, information technology, technology acceptance, medical informatics, informatics, health information technology, patient portal, personal health records, record, electronic health, personal health record, patient portals, email, health information systems, health information technology, mobile phones, information management, health information exchange, medical-records, e-health services, electronic medical-records, digitalization, data mining, electronic medical records, information systems, digital transformation, gamification, health records, teledermatology, remote consultation, technology adoption, medical records, nursing informatics,* and *tam* |
| 3 (Dark blue): eHealth technology, including privacy, security, and design | *management, hospitals* and *elderly people* | *diagnosis, medicine, monitoring, multiple sclerosis, medical services, chronic diseases, ecg, diseases, decision-support, tuberculosis, patient monitoring, personalized medicine,* and *home monitoring* | *e-health, iot, internet of things, cloud computing, blockchain, interoperability, artificial intelligence, smartphone, machine learning, big data, authentication, cloud, networks, protocol, m-health, architecture, encryption, internet of things (iot), sensors, mobile, sensor, access control, ehr, remote monitoring, fog computing, algorithm, 5g, algorithms, edge computing, electronic healthcare, deep learning, cryptography, network, privacy protection, smartphones, access control, platform, ehealth services, body area networks, wireless, health information management, attribute-based encryption, data protection, ontology, sensor networks, servers, wearable technology, activity recognition, ai, devices, e-health systems, transmission, wearables, patient monitoring, mobile-health, accelerometer, artificial intelligence (ai), data privacy, e-health system, electronic health record (ehr), mobile computing, ontologies, protection, wearable, wireless sensor networks,* and *connected health* |
| 4 (Yellow): eHealth literacy | *older adults, students, china, users, consumers, nursing students, african americans, college students, young adults, adolescence, consumer, chinese,* and *university students* | *health literacy, communication, covid-19, education, knowledge, health promotion, information-seeking, disparities, patient education, health education, health information-seeking, health disparities, readability, information seeking, awareness, online health information seeking, decision-making, misinformation, covid-19 pandemic, personalization, cyberchondria, health information seeking,* and *coronavirus* | *internet, ehealth literacy, online, internet use, social media, web, digital divide, e-health literacy, eheals, computer, digital health literacy, digital literacy, consumer health informatics, health technology, world-wide-web, website, electronic health literacy, social networks, instrument, assessment, online health information, computer literacy, web sites, facebook,* and *web 2.0* |
| 5 (Purple): health promotion and prevention of disease through active lifestyle choices | *adults, older-adults,* and *population* | *prevention, physical-activity, physical activity, exercise, obesity, disease, engagement, primary-care, overweight, pregnancy, weight-loss, hypertension, diet, health behavior, behavior-change, behavior change, smoking, type 2 diabetes, smoking-cessation, cardiac rehabilitation, cardiovascular-disease, smoking cessation, cardiovascular disease, diabetes mellitus, self-regulation, weight loss, childhood obesity, blended care, health-promotion, sedentary behavior, blood pressure, healthy lifestyle, self-monitoring, preventive medicine, behavior change, impairment, walking, cardiovascular diseases, health behaviors, lifestyle intervention, mellitus,* and *secondary prevention* | *ehealth interventions, mobile application,* and *user experience* |
| 6 (Light blue): mHealth^d^ and digital health | *elderly, aged,* and *society* | *primary care, chronic disease, rehabilitation, diabetes, medication adherence, self-care, asthma, copd, home, integrated care, stroke, heart failure, multimorbidity, inflammatory bowel disease, shared decision-making, arthritis, health equity, heart-failure, chronic obstructive pulmonary disease, equity, medication, screening, inequalities, obstructive pulmonary-disease, physiotherapy, rheumatology,* and *ulcerative-colitis* | *ehealth, mhealth, digital health, mobile health, mobile phone, user-centered design, apps, app, participatory design, telemonitoring, mobile applications, telerehabilitation, information and communication technology, mobile technology, health apps, virtual-reality,* and *application* |
| 7 (Orange): HIV prevention | *united-states, men, gay, young men,* and *african-american* | *decision-making, hiv, hiv prevention, sex, antiretroviral therapy, sexual health, behavioral intervention,* and *hiv/aids* | *acceptability, intervention development, digital,* and *digital technology* |

^a^ Cluster colors refer to Figure 2, A keyword co-occurrence network for 5,000 eHealth articles, interactive map: <https://tinyurl.com/2d7h2z87>.

^b^ Keywords from Figure 2 are shown in italics.

^c^ mHealth: mobile health.

# 2. Publication Impact: Mean Citations and Mean Normalized Citations

## Methods

The count of citations measure may penalize recent works that did not have much time to accumulate citations. Normalized citations control for publication recency, especially for studies that were accessible for at least a few months. Some studies in our corpus were published for only a few weeks.

We provided both citations and normalized citation overlays for keyword and concept co-occurrence maps for the following interactive maps available from Leiden University’s VOSviewer Online application.

A Keyword Co-occurrence Network for 5,000 eHealth Articles

- Mean citations overlay: <https://tinyurl.com/29493wfw>
- Mean normalized citations overlay: <https://tinyurl.com/2c6r68zr>

A Keyword Co-occurrence Network for 1,885 eHealth Reviews

- Mean citations overlay: <https://tinyurl.com/28wnqmur>
- Mean normalized citations overlay: <https://tinyurl.com/25h5xra8>

A Concept Co-occurrence Network for 10,022 eHealth Articles from OpenAlex

- Mean citations overlay: <https://tinyurl.com/25kh2dwf>
- Mean normalized citations overlay: <https://tinyurl.com/26v2na6q>

## Results

### Publication Impact: Mean Normalized Citations for Web of Science Articles and Reviews

To compare eHealth articles and reviews on mean normalized citations, we standardized this measure and computed delta for all keywords that are used to index no less than 50 articles or reviews. After we removed 3 non-specific keywords, the most cited articles, according to average normalized citations, were indexed with *internet of things, blockchain, childhood obesity, security, big data, risk-factors, gamification, pandemic, artificial intelligence,* and *aging.* With the exception of *gamification* and *pandemic*, the list of keywords for most cited reviews was different, likely reflecting the time gap between articles and reviews and the additional time needed to accumulate citations: *architecture, trust, health communication, older adults, consumer health information, web-based intervention, smoking-cessation, ehealth literacy,* and *user acceptance.* Interestingly, both study types had highly cited publications about aging or older adults.

Keywords that stood out as more likely to be associated with higher (> .5 SD) z-score converted normalized citation counts for reviews (vs. articles) were *feasibility, mental health, behavior, communication,* and *intervention*. On the other hand, articles indexed with *support, system, digital health, risk, quality-of-life,* and *adolescents* were more likely to accumulate citations than reviews indexed with the same keywords. However, this particular finding should be interpreted with caution due to reviews’ recency and the fact that it takes time accumulate citations.

Despite a plethora of publications, health literacy is somewhat unlikely to be the subject of reviews than other commonly reviewed eHealth topics. At the same time, eHealth literacy and data security are the most cited research areas for both eHealth articles and reviews, as indicated by normalized citations, a research impact measure that accounts for study recency. Highly cited eHealth reviews also involve the topics of mental health and cognitive behavioral therapy (CBT).

### Publication Impact: Mean Normalized Citations for eHealth Studies from OpenAlex

The distribution of mean normalized citations was overall similar to the distribution of publication years, with low scores observed for technology-oriented left side of the map and health topic on the right scoring high. Health literacy, obesity, and pandemic concepts stood out as particularly well-cited eHealth areas. Some technological concepts also scored high on normalized citations: *server, digital divide, internet access, wearable technology, blockchain, big data, smartwatch, internet of things*, and *wearable computer.*
